# Platinum-Based Catalysts on Various Carbon Supports and Conducting Polymers for Direct Methanol Fuel Cell Applications: a Review

**DOI:** 10.1186/s11671-018-2799-4

**Published:** 2018-12-22

**Authors:** Z. A. C. Ramli, S. K. Kamarudin

**Affiliations:** 1 Fuel Cell Institute, Universiti Kebangsaan Malaysia, 43600 Bangi, Selangor Malaysia; 2 Department of Chemical and Process Engineering, Universiti Kebangsaan Malaysia, 43600 Bangi, Selangor Malaysia

**Keywords:** Direct methanol fuel cell, Pt alloy, Pt-based electrocatalyst, Pt-transition metal, Carbon support, Conducting polymer support, Methanol electrooxidation

## Abstract

Platinum (Pt)-based nanoparticle metals have received a substantial amount of attention and are the most popular catalysts for direct methanol fuel cell (DMFC). However, the high cost of Pt catalysts, slow kinetic oxidation, and the formation of CO intermediate molecules during the methanol oxidation reaction (MOR) are major challenges associate with single-metal Pt catalysts. Recent studies are focusing on using either Pt alloys, such as Fe, Ni, Co, Rh, Ru, Co, and Sn metals, or carbon support materials to enhance the catalytic performance of Pt. In recent years, Pt and Pt alloy catalysts supported on great potential of carbon materials such as MWCNT, CNF, CNT, CNC, CMS, CNT, CB, and graphene have received remarkable interests due to their significant properties that can contribute to the excellent MOR and DMFC performance. This review paper summaries the development of the above alloys and support materials related to reduce the usage of Pt, improve stability, and better electrocatalytic performance of Pt in DMFC. Finally, discussion of each catalyst and support in terms of morphology, electrocatalytic activity, structural characteristics, and its fuel cell performance are presented.

## Introduction

Fuel cell technology has gained widespread attention around the world. Fuel cells (FCs) are a promising alternative power generation technology that converts chemical energy to electrical energy through an electrochemical reaction [1, 2]. Moreover, for fuel cell technology, the main focus in fuel cell technology is to generate low-cost production, thus achieving powerful performance of the fuel cell system and discovering durable materials. Nevertheless, the common issues that arise in current fuel cell technology are that the systems involve high intrinsic costs and poor durability [1]. Despite its promise as a fuel cell, direct methanol fuel cells (DMFCs) have challenges and limitations, leading researchers to study methods to improve the DMFC efficiency and performance. Many problems with DMFCs have been identified and remain unsolved, including crossover of methanol fuel from the anode electrode to the cathode electrode [3–5] poor performance caused by the slow kinetics rate, instability of the catalyst, and thermal and water management [6–8].

Recently, there have been numerous investigations on fuel cells, including DMFC, proton exchange membrane fuel cell (PEMFC), solid oxide fuel cell (SOFC), and so on, which are popular fuel cell technologies. As a novel energy source, DMFCs can be used for mobile and stationary applications [9, 10]. Many research advances have been achieved in the fuel cell field. Among the fuel cells, DMFCs have been extensively studied in recent years [11–16] because of their many advantages, such as high power density, ease of fuel handling, ease of charging, and low environmental impact [17, 18]. However, several technical challenges for the commercialization of DMFCs remain unresolved, including methanol crossover, low chemical reaction rates, and catalyst poisoning. However, DMFCs still have received attention from many researchers and have become the most popular fuel cells because of their low-temperature operation (DMFC systems operate at 373 K). Due to DMFC’s advantages of high energy efficiency and rapid start-up system, DMFC technology is very suitable to be applied as residential power sources, batteries in mobile devices, and as vehicle fuel [19–22]. In addition, the concept of DMFCs could be further studied to find alternative fuel sources such as from natural gas and biomass, as well as the fermentation of agricultural products to produce ethanol, in order to minimize the dependency on insecure energy sources [14].

In DMFC, anode side is supplied with methanol solution that will undergo electrooxidation to carbon dioxide (CO_2_) through the reaction below:

1 $$ {\mathrm{CH}}_3\mathrm{OH}+{\mathrm{H}}_2\to {\mathrm{CO}}_2+6{\mathrm{H}}^{+}+6{\mathrm{e}}^{\hbox{-} } $$

While at the cathode side the proton, the oxygen (from air) is reduced to water:

2 $$ 3/2\ {\mathrm{O}}_2+6{\mathrm{H}}^{+}+6\ {\mathrm{e}}^{\hbox{-}}\to 3{\mathrm{H}}_2\mathrm{O} $$

The net equation DMFC reaction can be summarized as follows:

3 $$ {\mathrm{CH}}_3\mathrm{OH}+3/2{\mathrm{O}}_2\to {\mathrm{CO}}_2+2{\mathrm{H}}_2\mathrm{O} $$

In DMFC systems, there are two types of DMFC modes: active and passive modes [23–25]. In an active DMFC system, the outlet stream of the DMFC stack is recirculated through the closed-loop control of the liquid methanol feed. Meanwhile, the liquid methanol at the anode stream is controlled by a methanol concentration sensor that plays an important role in providing sufficient injection of additional methanol and water to restore this fuel based on target concentration. There are several types of methanol concentration sensors that are used in the DMFC system to control and maintain the methanol feed concentration [17]. Usually, the liquid methanol is delivered to the anode side by a peristaltic pump while the surrounding air containing oxygen is supplied to the cathode side by a blower or fan [16]. In a passive mode of DMFC system, the liquid methanol is fed continuously to the system. This passive concept is very attractive for DMFCs system [26–28]. The concept of passive means the system operates totally autonomously without any support devices. The concept of passive DMFC means the system operates totally autonomously without any assistance of external device for pumping methanol and blowing air into the stack. In the passive mode of DMFC system, the catalyst layer will be supplied by methanol and oxygen as it reactants. During methanol oxidation reaction (MOR), CO_2_ and water will be removed out from the cell by passive means, i.e., diffusion, natural convection, capillary action, etc. [20]. Passive mode DMFC seems more advantageous compared to the active mode DMFC in terms of simpler, more compact design, and low cost. The complex system design and controls can be a disadvantage of active mode DMFC [21]. From the practical uses aspect, active mode DMFC appears to be more appropriate in high-power system, whereas the passive mode DMFC is more suitable to be used in low-power requirements [22].

**Fig. 1:**
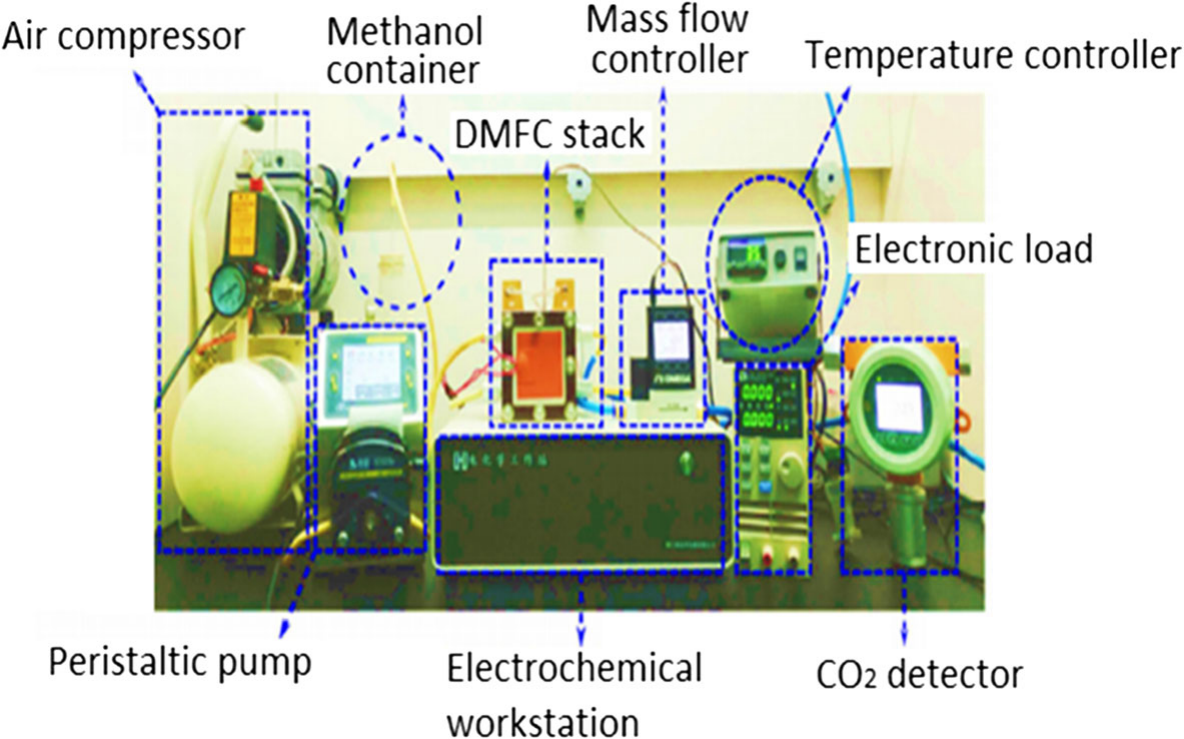
A general experimental set-up for single cell DMFC [23]

**Fig. 2:**
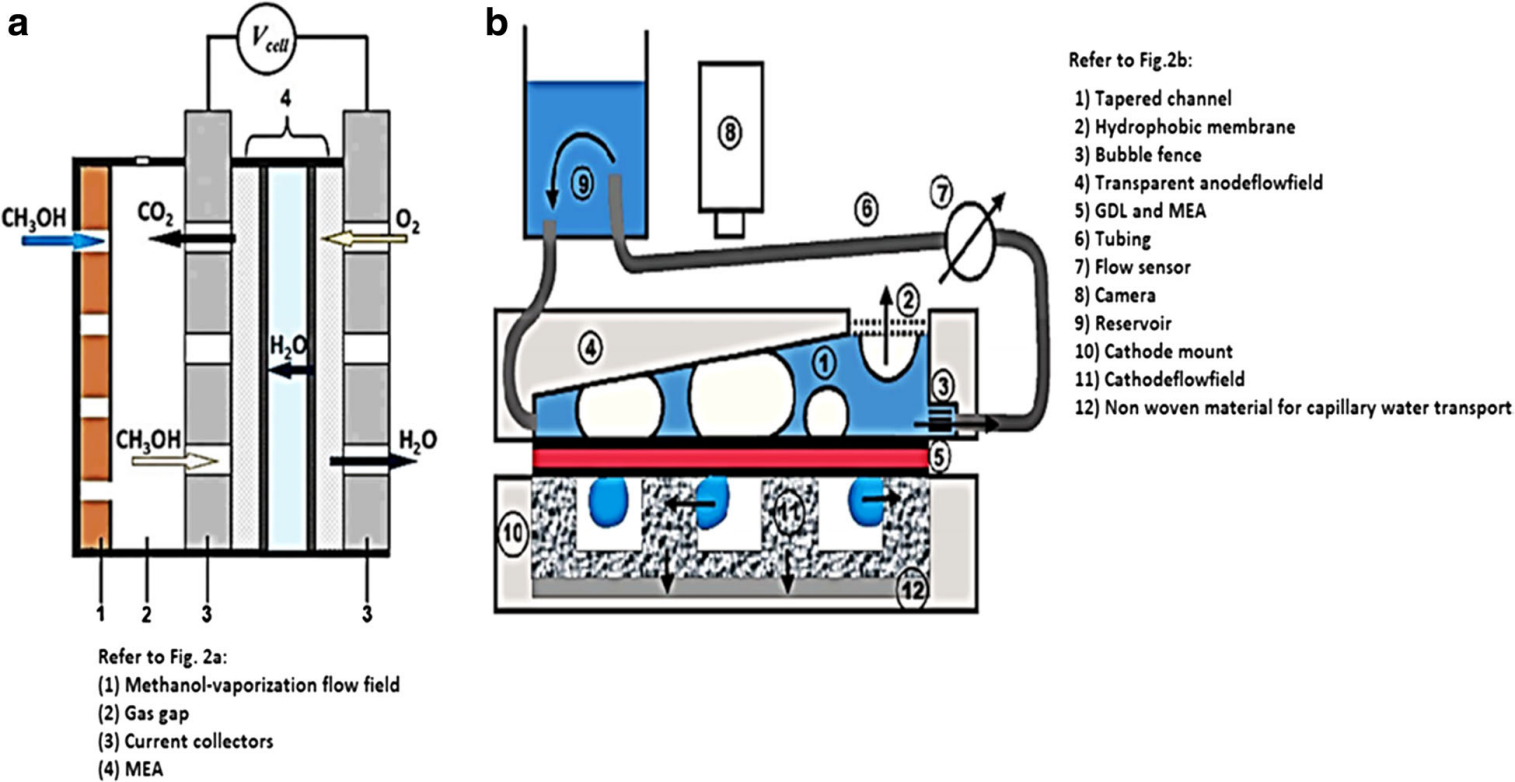
Schematic diagram of **a** active mode [24] and **b** passive mode [25] of DMFC

Figure 1 shows the set-up and design for a single-cell DMFC. A single-cell DMFC stack consists of a five-layer membrane electrode assembly (MEA) sandwiched by two plates which are anode and cathode. At the anode side, liquid methanol (contain methanol and deionized water) and absolute methanol is flowed into the channel by a peristaltic pump. At the cathode side, air is pumped into fuel cell by a rotameter. The temperature controller in DMFC stack is used to maintain the working temperature in the cell by a supplementary heating device. The electronic load device is used to change the current density to different levels and measure the corresponding values of the voltage. The cell performance is monitored by an electrochemical workstation, while the production of CO_2_ as final product of the overall reaction is measured by a CO_2_ concentration detector [23]. In direct methanol fuel cell, there are several importance operating parameters that must be considered during experimental study, which are (i) working temperature, (ii) methanol concentration, and (iii) input flow rates of feed methanol solution and air [23]. Figure 2a, b shows the DMFC’s active and passive mode, respectively.

This review will focus on the recent progress in the researches and developments of catalyst support based on Pt catalyst as the noble catalyst in DMFC. We include the activity of Pt-based catalysts combined with alloys, metals, transition metals, metal carbides, metal nitrides, and various carbonaceous species, such as graphene/graphene oxide (G/GO), carbon nanotube (CNT), carbon nanofiber (CNF), carbon nanocoil (CNC), carbon black (CB), multiwall carbon nanotube (MWCNT), and carbon mesoporous (CMS) supports, as well as conductive polymers, such as polyaniline (PANi) and polypyrrole (Ppy) as the support material. Many synthesis methods can be applied to prepare Pt-based catalysts. The most common methods applied to obtain nanoscale Pt particles are impregnation [29–34], hydrothermal techniques [35–41], microemulsion [42–45], and reduction [46, 47]. Generally, the preparation method could affect the morphology and size of the catalyst particles; thus, the selection of the method for catalyst synthesis is very important.

## Performance of Various Types of Pt-Based Catalysts

Over the past decade, many researchers have focused their research on the development of electrocatalyst in order to enhance its electrocatalytic activity in methanol MOR for DMFC system [37, 38]. Platinum (Pt) is a single-metal catalyst that shows significantly high catalytic activity for the MOR. However, pure Pt alone in a DMFC system may be easily poisoned by the intermediate species, i.e., carbon monoxide (CO), and the high cost of the Pt catalyst limits its commercial application as an electrocatalyst, thereby lowering the kinetic rate of methanol oxidation in DMFC system [48–50]. These three points are the main obstacles and limitations of using Pt alone as an electrocatalyst for DMFC. However, to overcome these hindrances, several studies have been conducted to synthesize Pt-based alloy electrocatalysts to achieve better electrocatalytic performance with less Pt usage [11, 47, 51, 52]. Normally, the average size of Pt particles and its morphology can be determined through scanning emission micrograph (SEM) or transmission electron micrograph (TEM) analysis, which are most common methods in catalysis field that can be used to characterize the physical properties of electrocatalysts. Table 1 shows the average particle sizes of the Pt particles with different of synthesis methods, properties, and their performances.

**Table 1:** Various type of Pt bimetallic, tertiary, and quaternary metal alloys and their performance

Type alloy	Particle size (Pt)	Preparation method	Structural/properties/performance	Ref.
PtRu	ND	Reduction	Pt–Ru (25:1) catalyst demonstrated highest electrocatalytic activity, higher resistance to CO, and better long-term stability compared to Pt–Ru (3:1), Pt–Ru (1:1), and Pt. Addition of more Ru could decrease the catalyst activities. CO species removed from catalyst surface by the OH at high potentials. Methanol electrooxidation activities also effected by of scan rate, temperature, methanol, and acid concentration.	[41]
PtRh	5.4 nm	Reduction	Synthesis of PtRh nanosponge (NS) with 3D porous structure with interconnected pores. Much more stable catalyst in methanol oxidation as proved by CA measurement. Greatly improved MOR activity as compared to Pt nanoparticles and commercial Pt/C catalyst. PtRh showed higher ECSA value than Pt nanoparticles.	[192]
PtAu	3 nm	Electrodeposition	From morphologies study Pt and Au, they are in spherical and dendrite shape. PtAu exhibited remarkably enhanced MOR (3.947 mA cm^−2^) The catalytic in MOR was 2.65 times higher that of commercial Pt/C (1.487 mA cm^−2^) which can be related with the electronic structure of Pt when Au surface was modified by fewer Pt nanoparticles. Much improved in poisoning tolerance.	[72]
PtSn	5.2 nm	Impregnation	The potential of PtSn/C-PANI was tested in a DMFC cell which showed lower methanol crossover by 30%. Higher in durability as compare to PtRu/C. Maximum current density observed 40% higher than PtRu/C. Highly CO tolerance.	[74]
PtNi	4.4 nm	Impregnation	[MOR]: Current density observed for PtNi/C catalyst is 5.3 times higher than commercial Pt20/C E-TEK. Charge transfer during MOR is facilitated for mixed oxides from the non-noble metal.	[55]
PtNi	2–3 nm	Polyol reduction	[MOR]: Heat treatment leads to segregation of Pt particles. Thus, lower MOR activity. Highest activity was found for a Pt to Ni atomic ratio of 3:1. Highly stable catalyst with addition of Ni. Mass specific activity over 200 potential cycles.	[77]
PtCo	2.4 nm	Chemical reduction	[MOR]: XPS analysis revealed the strong charge transfer interaction between Pt and Co atoms gives much higher electrocatalytic activity, stability and CO tolerance.	[64]
PtCo	2–5 nm	Chemical reduction	[MOR]: highest catalytic activity achieved by PtC ratio of 9:1. Exhibited three times power density as compared to commercial Pt/C catalyst. Efficient in methanol oxidation and CO adsorption on active Pt sites can be explained by bifunctional mechanism.	[51]
PtFe	0.7 nm	Chemical reduction	A higher Pt dispersion attributed to the temperature of chemical reduction route.	[52]
PtZn	3–5 nm	Microwave-assisted polyol	Stable electrochemical activity in acid medium and MOR Good Co tolerance and long-term durability. The higher performance attributed to the bifunctional mechanism of the binary catalysts: addition of Zn promotes the center for the generation of Zn–OH species, and more Pt sites are thus available for MOR.	[67]
PtRuSn	50 nm	Thermal decomposition	[MOR]: addition of Sn and Ru to the Pt increases the activity as described by bimetallic mechanism of bifunctional mechanism and electronic properties of Pt by contributing d-electron density in an electronic model.	[68]
PtRuNi	2.5–3.5 nm	Reduction	[MOR]: MOR for PtRuNi (1.98 mA/Cm^2^) was much as compared to PtRu (1.39 mA/cm^2^) and pure Pt (0.03 mA/cm^2^).	[69]
PtRuMo	2.06 nm		[DMFC]: ECSA = 138 m^2^ g^−1^_Pt_, mass activity = 15 A cm^2^ g^−1^_Pt_	[70]
PtNiCr	ND	Reduction	[MOR]: enhance in catalytic activity is attributed to the conditioning process caused dissolution and an oxidation state change of metallic Ni and Cr_2_O_3_ in the binary catalysts. The higher MOR of the ternary catalysts compared to the binary alloy was attributed to co-alloying of Ni and Cr, leads to expose more Pt surface sites without reducing specific activities of the catalysts. The binary and ternary catalysts result from both the well-known bifunctional mechanism and an electronic (ligand) effect.	[78]
PtRuOsIr	5–7 nm	Complex sol gel	[MOR]: composition of alloys: Pt-41 at.%Ru-10 at.%Os- 5 at.% Ir possess good chemical homogeneity and exhibited excellent catalytic activities.	[79]
PtRuIrSn	ND	Reduction	[MOR]: 25–35% Ir and 10% Sn content revealed high stability of catalyst, and higher in MOR activity than the commercially available E-TEK anode (80%[0.5Pt 0.5Ru]/C. Ir used is more than other co-alloy because it has higher stability than Pt and Ru. This composition can maintain high catalytic activity with low loading of Pt.	[81]
Pt/α-MoC	ND	temperature-programmed carburization (TPC)	α-MoC provides highly active sites for water dissociation. Produced abundant surface hydroxyl groups Methanol could be effectively activated (the effective barrier is 0.79 eV), water dissociation and the substantial reforming of CO cannot proceed at low temperatures (effective barrier of 1.18 eV). Well-dispersed Pt maximizes the exposed active interface of Pt1/α-MoC and effectively increases the density of active sites.	[82]

Bimetallic PtRu is considered as the most active catalyst due to its bifunctional mechanism and the ligand effects [48, 53]. PtRu becomes an interesting catalyst alloy, and has been used until nowadays with many carbon supports. However, the toxicological effect of the addition of ruthenium (Ru) metal remains uncertain [49]. Therefore, research on less expensive alloys that mix Pt with other nonprecious metals has been performed [49–52, 54–57], as discussed in “Performance of Pt-based alloys” section.

### Performance of Pt-Based Alloys

Arico et al. [35] found that numerous studies have been conducted to increase the catalytic activity of Pt catalysts in the MOR. In many studies, the optimal Pt-Ru ratio has been identified as 1:1, and particle sizes on the nanosized scale are the ideal size to improve catalyst utilization. However, Shi et al. [38] identified that 3:2 was the optimal ratio for Pt-Ru in their experiments to enhance MOR catalytic activity. Other than that, the electrocatalytic activity for methanol electrooxidation activity can also be increased if the PtRu electrocatalyst particles are nanosized particles in the range of 2–4 nm. Paulas et al. [39] agreed with this statement. As we know, Pt shows high reactivity toward methanol fuel, rendering Pt metal as an ideal electrocatalyst for the anode electrode in DMFC system. Nevertheless, during MOR of Pt catalyst, carbon monoxide (CO), i.e., the intermediate species, will form on the surface of Pt particles, which thus poisons the catalyst surface [58–61]. Thus, some efforts are needed to overcome the problem related to the formation of poisonous species on the Pt particles surface, so that they do not cover up the active site areas of Pt. Generally, binary alloys, such as PtRu [62–66], PtRh [67–71], PtAu [72–74], PtSn [62, 63, 75–77], PtNi [64–69], PtCo [70, 71, 78–80], and PtFe [81–85], are frequently employed as electrocatalyst combinations for the anode electrode in DMFC system. The additions of these metals, such as ruthenium (Ru), tin (Sn), and rhodium (Rh), are believed to produce higher catalytic activity.

Incorporation of Nickel (Ni) into platinum-based catalyst gives superior performance for MOR and DMFC. In latest research, Guerrero-Ortega and co-workers explain the addition of Ni in the Pt-Vulcan support promotes an important increment in the faradic current during MOR of one order of magnitude, even though the use Pt is lower in the bimetallic catalyst [55] . Their experimental results also suggested that the addition of Ni promotes some structural and electronic modifications that enhance a better reaction performance at the electrode interface. In another work, incorporation of Au into Pt alloy enhanced the electrocatalytic activities because of the changing electronic structure and the improvement of the electrochemically active area (ECSA) [47]. While, the addition of Tin (Sn) to Pt-based alloy showed an increment in electrocatalytic activity, which is strongly influenced by the incorporation of Sn in its alloy system and oxidized forms, boosting the reaction more readily because of the lower oxidation potential [56]. Also, the addition of Cobalt (Co) to Pt-based alloy improved the catalytic properties greatly by PtCo (1:9)/rGO catalyst that found to be ten times higher than Pt/rGO [51]. The increase in current density is attributed to higher dispersion of PtCo nanoparticles on hydrophilic nature of rGO support that promotes water activation and leads to oxidize the CO_ads_ on Pt sites. Furthermore, according to the bifunctional mechanism of Co, it promotes the H_2_O activation creating more -OH ions and other O_2_-containing species to oxidize CO- intermediates species on Pt site [57]. This bifunctional mechanism of Co could also be used for other catalytic transition metals toward MOR. The catalytic oxidation mechanism for CO species to CO_2_ in the presence of PtCo catalysts can be summarized as follows:

4 $$ \mathrm{Pt}+{\mathrm{CH}}_3\mathrm{OH}\to \mathrm{Pt}\hbox{-} {\mathrm{CO}}_{\mathrm{ads}}+4{\mathrm{H}}^{+}+4{\mathrm{e}}^{\hbox{-} } $$

5 $$ \mathrm{Co}+{\mathrm{H}}_2\mathrm{O}\to \mathrm{Co}{\left(\mathrm{OH}\right)}_{\mathrm{ads}}+{\mathrm{H}}^{+}+{\mathrm{e}}^{\hbox{-} } $$

6 $$ {\mathrm{PtCO}}_{\mathrm{ads}}+\mathrm{Co}{\left(\mathrm{OH}\right)}_{\mathrm{ads}}/{\mathrm{CO}}_2+\mathrm{Pt}+\mathrm{Co}+{\mathrm{H}}^{+}+{\mathrm{e}}^{\hbox{-} } $$

Furthermore, Löffler et al. [86] successfully synthesized PtRu as the anode catalyst for DMFCs by which produced the most active electrocatalyst for methanol electrooxidation at approximately 50 at.% Ru. Meanwhile, Dinh et al. reported [87] that PtRu with ratio of PtRu 1:1 have a stronger metallic behavior and higher electrocatalytic activity for methanol oxidation (MOR). The performance are related to these two major factors: (i) maximized catalyst surface area and (ii) catalyst surface with maximum number of metal alloy sites of atomic ratio close to 1:1. Both of these group also showed highly. Based on the bifunctional mechanism, Aricò et al. [58] and Goodenough et al. [62] suggested that the CO intermediate species that formed on the Pt surface-active sites can be oxidized to carbon dioxide (CO_2_) by active oxygen atoms formed on the secondary elements, for example Ru, Sn, and Mo, in the lower potential region. Table 1 summarized the performance of various type of Pt alloy catalyst carried out by researchers for MOR. According to bifunctional mechanism [88–90], the MOR on supported PtRu alloy catalysts can be summarized as the following equation. Pt is a more active catalyst for the methanol adsorption than Ru. Hence, the overall reaction on PtRu electrocatalysts for methanol oxidation reaction obeys the bifunctional mechanism.

7 $$ \mathrm{Pt}+{\mathrm{CH}}_3\mathrm{OH}\to \mathrm{Pt}\hbox{-} {\mathrm{CH}}_3\mathrm{OH}\mathrm{ads}\to \mathrm{Pt}\hbox{-} {\mathrm{CO}\mathrm{H}}_{\mathrm{ads}}\to 3\mathrm{H}+3\mathrm{e}\hbox{-} \to \mathrm{Pt}\hbox{-} {\mathrm{CO}}_{\mathrm{ads}}+{\mathrm{H}}^{+}+{\mathrm{e}}^{\hbox{-} } $$

8 $$ \mathrm{Ru}+{\mathrm{H}}_2\mathrm{O}\to \mathrm{Ru}\hbox{-} {\mathrm{OH}}_{\mathrm{ads}}+{\mathrm{H}}^{+}+{\mathrm{e}}^{\hbox{-} } $$

9 $$ \mathrm{Pt}\hbox{-} {\mathrm{CO}\mathrm{H}}_{\mathrm{ads}}+\mathrm{Ru}\hbox{-} {\mathrm{OH}}_{\mathrm{ads}}\to \mathrm{Pt}+\mathrm{Ru}+{\mathrm{CO}}_{2+}2{\mathrm{H}}^{+}+2{\mathrm{e}}^{\hbox{-} } $$

10 $$ \mathrm{Pt}\hbox{-} {\mathrm{CO}}_{\mathrm{ads}}+\mathrm{Ru}\hbox{-} {\mathrm{OH}}_{\mathrm{ads}}\to \mathrm{Pt}+\mathrm{Ru}+{\mathrm{CO}}_2+{\mathrm{H}}^{+}+{\mathrm{e}}^{\hbox{-} } $$

Referring to this bifunctional mechanism, methanol is initially dissociated and adsorbed on Pt, subsequently decomposed to CO_ads_ and/or formyl-like species -CHO_ads_ by dehydrogenation reaction (7). At the same time, water dissociates to OH_ads_ and adsorbed on Ru sites (8). Then the species adsorbed on Pt and Ru sites and combine together to form CO_2_ molecule (9) and (10). The reaction between Pt–CO_ads_ and Ru–_OHads_ leads to CO_2_ evolution, generating refreshed Pt and Ru sites (reaction 10). Whereas, another work done by Ewelina Urbanczyk et al. [48] carried out the methanol oxidation reaction for PtNi catalyst in alkaline medium (1.0 M KOH). Theoretically, the methanol oxidation reaction in alkaline medium is:

$$ {\mathrm{CH}}_3\mathrm{OH}+6\mathrm{OH}\to {\mathrm{CO}}_2+5{\mathrm{H}}_2\mathrm{O}+6{\mathrm{e}}^{\hbox{-} } $$

The reaction initiates at Pt electrode of the DMFC to carbon dioxide. During this process, intermediate molecules (CO), may form that can cause poison and deactivation of the Pt active side. This CO molecule is a product of incomplete oxidation of methanol. The incomplete methanol oxidation forms the CO as the intermediate product (Eq. 11). The electrocatalyst surface can also adsorb hydroxyl groups (Eq. 6). Finally, due to the desorption of the main product, carbon dioxide is formed (13). The second poison which may be produced during the methanol oxidation is methane. In this case, the following reaction can occur (8). The total oxidation of the intermediate form of carbon to carbon dioxide in the electrochemical reaction is as follows:

11 $$ 3\mathrm{Pt}+{\mathrm{CH}}_3\mathrm{OH}\to \mathrm{Pt}-\mathrm{COads}+4{H}^{+}+2\mathrm{Pt}+4e-+{H}^2O $$

12 $$ \mathrm{Ni}+{H}_2O\to \mathrm{Ni}-\mathrm{OHads}+{H}^{+}+e- $$

13 $$ \mathrm{Pt}-{\mathrm{CO}}_{\mathrm{ads}}+\mathrm{Ni}-\mathrm{OHads}\to {\mathrm{CO}}_2+{H}^{+}+\mathrm{Pt}+\mathrm{Ni}+e- $$

14 $$ \mathrm{Pt}-{\mathrm{CH}}_3+\mathrm{Pt}-H\to 2\ \mathrm{Pt}+{\mathrm{CH}}_4 $$

15 $$ \mathrm{Pt}-{\mathrm{CH}}_3+\mathrm{Ni}{\left(\mathrm{OH}\right)}_2\to \mathrm{Pt}+{\mathrm{CO}}_2+\mathrm{Ni}+5{H}^{+}+5e- $$

Currently, researchers are still studying alloying techniques to improve the catalytic activity of Pt-based electrocatalysts by fabricating ternary and quaternary alloys of Pt, such as PtRuSn [91, 92], PtRuNi [93–95], PtRuMo [70, 96, 97], quaternary PtRuOsIr [79, 80], and PtRuIrSn [97, 98], because of their excellent behavior in MOR and removal of the intermediate species (CO) that form on the Pt surface site. But, the addition of the third and fourth metals in these ternary and quaternary catalysts is still unknown. Moreover, there are some limitations and challenges in producing the ternary and quaternary alloys. The optimization of catalyst morphology and catalyst compositions becomes difficult to be obtained because of many possible combinations of metals and compositions. However, many studies proved that the addition of third and fourth metal remarkably enhanced the catalytic activity, increase stability of catalyst, and good CO tolerance toward methanol electrooxidation and DMFC applications.

**Fig. 3:**
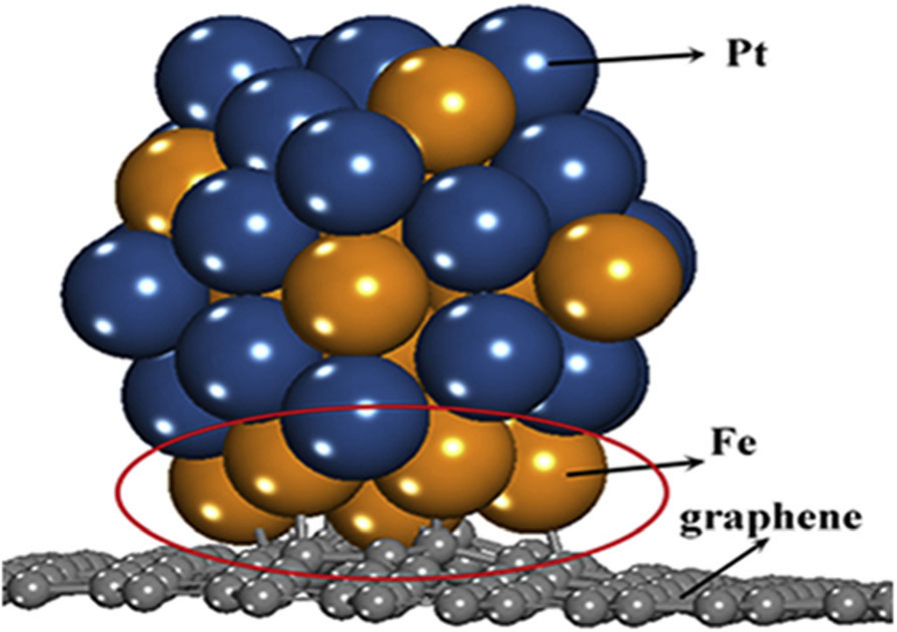
The position of PtFe catalyst on graphene support illustrated by Yang et al. [101]

Tsiouvaras et al. [99] conducted the electrochemical measurement of PtRuMo/C catalysts and found that although all ternary catalysts were more active toward CO and methanol oxidation than the binary catalyst, the catalyst treated with H_2_ showed an improved performance by approximately 15% with respect to the ternary catalysts treated in He or with no treatment. In 2012, Hu et al. [100] successfully synthesized a superior bimetallic (PtNi) electrocatalyst, namely hollow mesoporous PtNi nanospheres (HMPNNs). The catalyst exhibited outstanding catalytic performance in the MOR with significantly enhanced Pt utilization efficiency because of the unique structure of HMPNNs and their large electrochemical surface area. While around 2016, work done by Yang et al. [101] also investigated the reactivity of synthesized bimetallic PtFe electrocatalysts in which they observed that the strong interaction between Pt and iron (Fe) metals can decrease adsorption energies of bimetallic NPs. They also found that the bimetallic PtFe nanoparticles prefer to be adsorbed on the single vacancy graphene through the Fe atoms when Pt and Fe atoms are both on the surfaces, because the interactions between Fe atoms and single vacancy graphene is stronger than those between Pt atoms and single vacancy graphene. Figure 3 illustrates the position of Pt and Fe particles dispersed on the graphene support as suggested by Yang et al. [101].

### Performance of Pt-Based Catalyst and Transition Metal Carbide

Transition metal carbide (TMC), with high mechanical and chemical stability against corrosion, good resistance to acidic environments, long-term stability, and high CO tolerance, can act as anode catalysts [88–90, 102–104]. In addition, TMCs also provide many advantages compared to their parent metals with respect to the activity, selectivity, and resistance to poison, for example tungsten carbide (WC) shows special properties, such as good electrical conductivity, resistance to acidic environments, low cost, and tolerance to CO poisoning in methanol electrooxidation process [88, 105, 106].

Wang et al. [103] reported the synthesis of high surface area (256 m^2^ g^−1^) tungsten carbide microspheres via a simple hydrothermal method. W_2_C was found as the main phase in the as-synthesized sample. Currently, researchers are currently exploring the potential of Pt supported on WC as an ideal catalyst for DMFC [38, 88, 107, 108]. Christian et al. [106] concluded that relative to their transition metal elements, TMCs behave such as noble metals such as Pt, Pd, Rh, and Ru for certain chemical and electrochemical reactions, including oxidation reaction of hydrogen, carbon monoxide, and alcohol and reduction of oxygen [109, 110]. In another study, Liu et al. [107] presented that molybdenum carbides (Mo-Carbides) could act as promoters for tungsten carbide and increase the electrocatalytic activity in the DMFC. However, without the incorporation of Pt metal, the electrocatalytic activity of pure WC toward the MOR for a DMFC system is still low. Therefore, a small amount of platinum metal added onto the WC component is very convenient to gain the advantage of the synergetic effect between Pt and WC [91, 111, 112]. Meanwhile, Hassan et al. [109] revealed that the common impurity (CO species) that forms during methanol oxidation has strong binding energy on the Pt surface; therefore, it must be oxidized so that it can be removed from the Pt active sites. The addition of WC component in Pt/WC electrocatalyst has shown high CO tolerance for MOR, which indicates the existence of synergetic effects between the Pt metal and WC as the support component. The study was also performed by another researcher by using less Pt metal to reduce the cost of Pt, while maintaining good electrocatalytic performance.

Other than that, the WC component is more active for the formation of a methoxy group (CH_3_O-) than pure Pt [113, 114]. CV performance of (Pt:Ru)4-WC/RGO shows outstanding catalytic performance with a current density of 330.11 mA mg^−1^ Pt compared to the other five catalysts, indicating that the as-synthesized electrocatalyst has excellent catalytic activity toward the MOR. In addition, the combination of Ru and WC on the Pt catalyst increased the OH surface amount and allowed the CO adsorbed on the Pt surface to be oxidized at lower potentials [39].

### Performance of Pt-Based Catalyst and Transition Metal Nitride

Transition metal nitride (TMN) is an ideal candidate as a Pt catalyst support due to its good electrical conductivity (metallic), hardness, high electrochemical stability, and corrosion resistance under fuel cell operating conditions [115–118]. Transition metal nitrides, such as CrsN, TiN, and VN, supported Pt catalyst have been reported and showed high catalytic performance and better stability compared to the traditional carbon supports [112]. All transition metals can form nitride, except the second and third row of group 8, 9, and 10 metals (Ru, Os, Rh, Ir, Pd, and Pt). The behavior and structural properties of transition metal nitrides can be found in the literature [92–94]. Xiao et al. [112] prepared a titanium cobalt nitride-supported Pt electrocatalyst which showed excellent performance and stability toward the oxygen reduction reaction (ORR). The Ti_0.9_Co_0.1_N-supported Pt catalyst exhibited a small particle size and good metal dispersion. This prepared electrocatalyst also maintained the electrochemical surface area (ECSA) of Pt and greatly improved the ECSA preservation, with only a 35% decrease in the early ECSA drop after 10,000 ADT cycles. Cobalt doping significantly enhanced the ORR activity and durability. Meanwhile, a high performance and durable electrocatalyst in the DMFC system can be obtained using large surface area Pt(Ru)/TiN electrocatalyst, which also demonstrated high electrochemical activity toward MOR with a ∼ 52% improvement of catalytic activity and good stability/durability compared to commercial JM-Pt(Ru). Meanwhile, the DMFC’s single-cell performance achieved better maximum power density by 56% and demonstrated outstanding electrochemical stability for CSG-Pt(Ru)/TiN electrocatalyst [115].

Current research on Pt nanoparticles supported on titanium iron nitride nanotubes with hollow and porous structure and high surface area was synthesized by Li et al. [116]. It exhibited a significant increase in the electrocatalytic activity toward the MOR in acidic condition and had better durability. The reasons for these properties were due to their experimental data work that verified the addition of Fe can tune the electronic structure of Pt atoms, which contributes to the strengthened activity and stability of the Pt catalyst for the MOR. Meanwhile, in previous work done by Xiao et al. [117], Pt/Ti_0.8_Mo_0.2_N catalyst exhibited a porous structure and high surface area, small-size and well-dispersed Pt nanoparticles. This catalyst system preserved the intrinsic electrochemical stability of the TiN nanostructure and remarkably enhanced the MOR activity and durability. However, the currently available information on the electrochemical stability of tungsten nitride (WN) is still insufficient [109].

Meanwhile, MoxN (*x* = 1 or 2) on Ti substrate displayed electrochemical stability in an acidic electrolyte of 4.4 M H_2_SO_4_ up to anodic potential of + 0.67 V (vs. SHE) over 50 repeated cycles [110]. However, this electrocatalyst showed surface damage, such as cracking and crumbling, in the high cathodic (below − 0.1 V vs. SHE) and anodic (above + 0.67 V vs. SHE) potential regions due to cathodic and anodic corrosion, respectively. In the high anodic potential region above + 0.67 V (vs. SHE), the composition of oxygen increased due to MoOx oxide formation, which could cause deactivation. These results indicate that MoxN reacts with the oxygen species present in the aqueous electrolyte and is unstable above + 0.67 V (vs. SHE). Mustafha et al. [111] found that Pt loaded onto TiN as a support showed electroactivity for the methanol oxidation, with a high If/Ib ratio representing high CO resistance in the voltammogram performed at a scan rate of 20 mV/s in 0.5 M CH_3_OH + 0.5 M H_2_SO_4_ as the electrolyte. The bifunctional effect between Pt and TiN was cited as the cause of the CO resistance of Pt/TiN. In addition, Ottakam Thotiyl et al. [91] achieved good results for a Pt-loaded TiN catalyst, showing very good CO tolerance for the electrochemical oxidation of methanol. They concluded that the special characteristics of TiN that made it suitable as a Pt support for the MOR in an alkaline medium are that it shows exceptional stability, extreme corrosion resistance, good electronic conductivity, and strong adhesion behavior. TiN-supported catalysts are beneficial in terms of long-term stability, exchange current density, and stable currents at low overpotential. Platinum loadings of 40 wt% on TiN were used in the experiments.

In recent years, Liu et al. [118] successfully synthesized platinum on titanium nickel nitride decorated 3D carbon nanotubes which reduced graphene oxide (TiNiN/CNT-rGO) support by solvothermal process followed by nitriding process. Pt with small particle size is well-dispersed on TiNiN/CNT-rGO support. The 3D shape of CNT-rGO support gives a fast route for charge transfer and mass transfer as well as TiNiN NPs with good synergistic effect and the strong electronic coupling between different domains in TiNiN/CNT-rGO support. Thus, it greatly improved the catalytic activity of this catalyst. In another research, the non-carbon TiN nanotubes-supported Pt catalyst done by Xiao et al. [119] also displayed enhanced catalytic activity and durability toward MOR compared with the commercial Pt/C (E-TEK) catalyst.

### Performance of Pt-Based Catalysts with Transition Metal Oxide

Pan et al. [92] reported the synthesis of platinum–antimony-doped tin oxide nanoparticles supported on carbon black (CB) as anode catalysts in DMFC, which exhibited better improvement in catalytic activity toward MOR compared to Pt-SnO_2_/C or commercial Pt/C electrocatalyst. The enhancement in activity was attributed to the high electrical conductivity of Sb-doped SnO_2_, which induced electronic effects with the Pt catalysts. Another work done by Abida et al. [93]described the preparation of Pt/TiO_2_ nanotube catalysts for methanol electrooxidation. The TiO_2_ nanotubes-supported Pt catalyst (Pt/TiO_2_ nanotubes) exhibited excellent catalytic activity toward MOR and had good CO tolerance. They also reported that the use of hydrogenotitanate nanotubes as a substrate for the Pt catalyst considerably improved the CO_ads_ oxidation on Pt, but the MOR still occurred at high potential. Then, several years later, Wu et al. [94] synthesized Pt-C/TiO_2_ with MOR activity 1.6 higher than commercial Pt-C and the stability of Pt-C/TiO_2_ was also enhanced by 6.7 times compared to Pt-C. The excellent performance of this catalyst was a contribution of mesopores and partially coated carbon support. Zhou et al. [95] prepared hollow mesoporous tungsten trioxide microspheres (HMTTS) using the spray-drying method to yield Pt/HMTTS. The electrocatalyst exhibited excellent electrocatalytic activity and high stability toward MOR than Pt/C and Pt/WO_3_ electrocatalysts, which may be attributed to the well-ordered Pt particles (with an average size of 5 nm) on the HMTTS surface. Wu et al. [120] used polystyrene spheres as templates to obtain pore-arrayed WO_3_ (p-WO_3_). The Pt nanoparticles with an approximate size of 3.3 nm dispersed on pore-arrayed WO_3_ (Pt/p-WO_3_) exhibited high catalytic activity toward MOR.

Li et al. [121] used Sn-doped TiO_2_-modified carbon-supported Pt (Pt/Ti_0.9_Sn_0.1_O_2_–C) as an electrocatalyst for a DMFC system. The synthesized Pt/Ti_0.9_Sn_0.1_O_2_–C electrocatalyst revealed high catalytic activity and CO tolerance toward MOR. The enhanced catalyst activity was due to the high content of OH groups on the Ti_0.9_Sn_0.1_O_2_ electrocatalyst sample and the strengthened metals and support interactions. In addition, Lv et al. [122] also reported in their work that the addition of TiO_2_ could not only facilitate CO removal and hinder CO formation on Pt surface during methanol oxidation, but it can also prevent the agglomeration and corrosion of Pt, which can be concluded from strong metal-supports interaction between TiO_2_–C and Pt. Huang et al. [123] revealed that a TiO_2_-coated carbon nanotube support for Pt electrocatalysts could be prepared via a one-step synthesis. Hao et al. [124] developed a new catalyst composed of Pt nanoparticles deposited on graphene with MoO_3_. These catalysts exhibited high catalytic activity toward MOR and high resistance to CO species. However, the size of MoO_3_ must be tuned by controlling the metal oxide loading.

The selection of metal oxide such as MnO, RuO, CeO, SnO_2_, MgO, and V_2_O_5_ as additional component in electrocatalyst of Pt because of their low cost, good electrochemical properties, and have proton-electron intercalation properties [125]. From the catalytic activity aspect, it can be summarized that the addition of these metal oxides can enhance the electrocatalytic activity of DMFC and other fuel cells. The incorporation of these conducting metal oxides together with Pt catalyst could also facilitate the oxidation process of CO intermediate molecules. Hence, these types of metal have high potential to be used together with platinum as anode electrode.

## Carbon support

To improve the utilization of the Pt catalysts, the carbon support is also another useful approach to be used together with Pt. Carbon materials are largely used as catalyst support because of its special properties such as relatively stable in both acid and basic electrolyte, good conductivity, and provide high surface area for dispersion of metal catalyst. It is believed that carbon materials have a strong effect that can influence the electrocatalysts properties such as metal particle size, morphology, metal dispersion, alloyed degree, and stability. Carbon supports can also affect the performance of supported catalysts in fuel cells, such as mass transport and catalyst layer electronic conductivity, electrochemical active area, and metal nanoparticle stability during the operation.

Currently, a great concern of the development in the nanotechnology field, especially carbon nanomaterials synthesis, is to create more stable and active supported catalysts. Support materials of nanoparticles are believed to be the most promising materials for catalytic activity in fuel cells, including the DMFC system. Pt has been traditionally used as nobel catalysts for many fuel cells application [126–128]. However, the high cost and low reserve are hindering commercialization of fuel cells and driving researchers to make the utmost of the catalyst. According to this problem, the major effort has been done toward nanoscaling of the catalyst nanoparticles to form more active sites per mass unit. The morphology, structure, and activity of the catalyst, and correspondingly the whole lifetime of a cell, thus strongly depend on the catalyst support [129]. Table 2 shows the preparation, physical properties, performance, and activity of Pt-based supported carbon done by groups of researchers. The details of Pt-based supported carbon will be performed in the following sections: “Graphene Support” to “Carbon Nanocoils”.

**Table 2:** summarize the preparation, physical properties, performance, and activity of Pt-based supported various carbon materials

Catalyst	Preparation of support	Ave. particle size (nm)	Electrochemical condition	Catalytic properties	Advantages/limitations	Ref.
Pt/graphene nitrogen doped carbon layer (Pt/NCL-RGO)	Hummer’s method	3–6	0.5 M H_2_SO_4_ + 0.5 M CH_3_OH, at 25 °C, scan rate 100 mV/s	[MOR]: a better catalytic activity and stability. Peak current density of Pt/NCL-RGO is almost twice of Pt/RGO.	[Adv.]: NCL from aniline source prevented the aggregation of Pt on graphene nanosheet, larger ESA of Pt/NCL-RGO. [Limitations]: introduction of aniline contributed little effect on the crystallization of Pt particles.	[146]
PtRu/graphene	Hammer’s method	Less than 10	0.5 M H_2_SO_4_ + 1.0 M CH_3_OH, scan rate 50 mV/s	[MOR]: Current density of MOR for Pt/G 19.1 mA/cm^2^and Pt/CB is 9.76 mA/cm^2^, with the ratios are 6.52 for Pt/G and 1.39 for (Pt/CB), respectively.	[Adv]: graphene supported Pt behave a more stable fashion than those Pt on carbon black. Addition of Ru inhibit the accumulation of CO and other carbonaceous species formed on the graphene [Limitation]: the synthesis method need to be modified the get more uniform PtRu particles.	[193]
Pt/C/graphene aerogel	Green hydrothermal	2	0.5 M H_2_SO_4_ + 1.0 M CH_3_OH, scan rate 50 mV/s	[MOR]: gives higher stability during MOR. Current density observed is 405.3 mA mg^−1^ Pt, which is very close to that commercial of Pt/C (424.6 mA mg^−1^ Pt). Pt/C/GA more stable than Pt/C for methanol oxidation.	[Adv]: 3D macroporous structure of graphene support gives higher stability during MOR much affected by the hydrothermal process. 3D microporous graphene provide accessibility for reactant to the Pt NPs to ensure an effective mass transfer.	[144]
Pt/MWCNT	Reduction by using Ethylene glycol (EG)	3.4	1.0 M CH_3_OH, at 90 °C operating temperature, flow rate: 1.0 mL/min; oxygen pressure: 0.2 MPa	[ORR]: Catalytic activity of Pt/MWCNT is higher than that Pt/XC-7. Current density for Pt/MWCNT was 14.7 mA/mg Pt and 2.5 mA/mg Pt for Pt/XC-72.	[Adv]: increased of ORR activity of Pt/MWCNTs attributed to the unique structure, good electrical and electrical conductivity of MWCNT supports. Homogeneous dispersion of the Pt particles on support when the EG solvent is used, in contrast to aqueous HCHO reduction [Limitation]: particle size of Pt in Pt/MWCNT larger than those in Pt/XC-72 using this method.	[194]
PtFe/MWCNT	Reduction	1.5–2.1	0.5 M H_2_SO_4_ + 1.0 M CH_3_OH, at 25 °C, under N_2_ flow, scan rate 100 mV/s	[MOR]: S3 sample (PtFe/MWCNT) showed highest current density (86 mA/cm^2^) Improvement in CO tolerance	[Adv]: enhanced in electroactivity of S3 sample was due to the presence of Fe atoms on the surface of the Pt nanoparticles, that promotes a shift in the oxidation potential [Limitation]: ununiform particles size observed on carbon support.	[195]
PtRuNi/MWCNT	Purchased	2–4	0.5 M H_2_SO_4_ + 2.0 M CH_3_OH, scan rate 100 mV/s	[MOR]: current density achieved was 4000 mA/mg_Pt_. Highest tolerance to CO and more complete oxidation of methanol to CO_2_ in the forward scan.	[Adv]: addition of Ni in the PtRu can be explained by the hydrogen spillover effect of Ni hydroxides and electron effect of metallic. [Limitation]: difficulty in achieving uniform alloy formation and controlling the metal alloy composition on support.	[126]
PtRu/SWCNT	Chemical vapor deposition	2–3.5	0.5 M H_2_SO_4_ + 1.0 M CH_3_OH, at 25 °C, scan rate 50 mV/s	[MOR]: bimetallic catalyst supported on the different SWCNT buckypapers have excellent catalytic activity MOR.	[Adv]: higher in MOR was influenced by the solvent/dispersant and the presence of surface oxygen functional groups [Limitation]: the large agglomeration of particles also determined in range 12–15 nm.	[127]
PtRu/CECNF	Electrospinning	3.0	0.5 M H_2_SO_4_ + 0.5 M CH_3_OH, at 25 °C, scan rate 20 mV/s	[MOR]: PtRu supported CECNF exhibited 2.5 times higher in power density with one half the PtRu loading compared to that of the PtRu/C	[Adv]: higher in MOR attributed to the strong interaction of PtRu alloy and CECNF support. High oxygen storage capacity of CeO_2_ [Limitation]: CeO_2_ has characteristic of low electrical conductivity as support causes a high resistance which is a disadvantage for fuel cell application.	[196]
PtCo/CNF	Electrospinning	ND	0.5 M H_2_SO_4_ + 0.5 M CH_3_OH, at 25 °C, scan rate 20 mV/s	[MOR]: high catalytic activity MOR. More stable catalyst for the Pt/Co-coal-CF compared to that Pt /CNF, coal-based carbon nanofiber (coal-CF) and Co embedded carbon nanofiber (Co-CF). Mass activity of PtCo-coal-CF 78.5 A/g Pt.	[Adv]: high graphitization of Co-coal-CF was obtained. Electrospinning have drawn the most advantages of simple method, low price, high yield and easy morphology control of particles. Pt nanoparticles are well distributed on the coal-CF, Co-CF, and Co-coal-CF materials. [Limitation]: agglomeration of nanoparticle clusters are still observed, that may because of weak interaction between Pt nanoparticles and CF support.	[131]
PtCo/wormlike mesoporous carbon	Reduction	3–4	0.5 M H_2_SO_4_ + 0.5 M CH_3_OH, at 25 °C, scan rate 50 mV/s	[MOR]: 26–97% increase in catalytic activity than that of commercial catalyst. BET surface area obtained of GMC is 585 m^2^ g^−1^.	[Adv]: high in degree of graphitization with 2-D hexagonal mesoporous structure exhibited high capacitance and conductivity of this support. Reduce the usage of Pt. Low temperature used [Limitation]: the specific surface area of mesoporous carbon not much larger and need to be improved.	[197]

### Graphene support

**Fig. 4:**
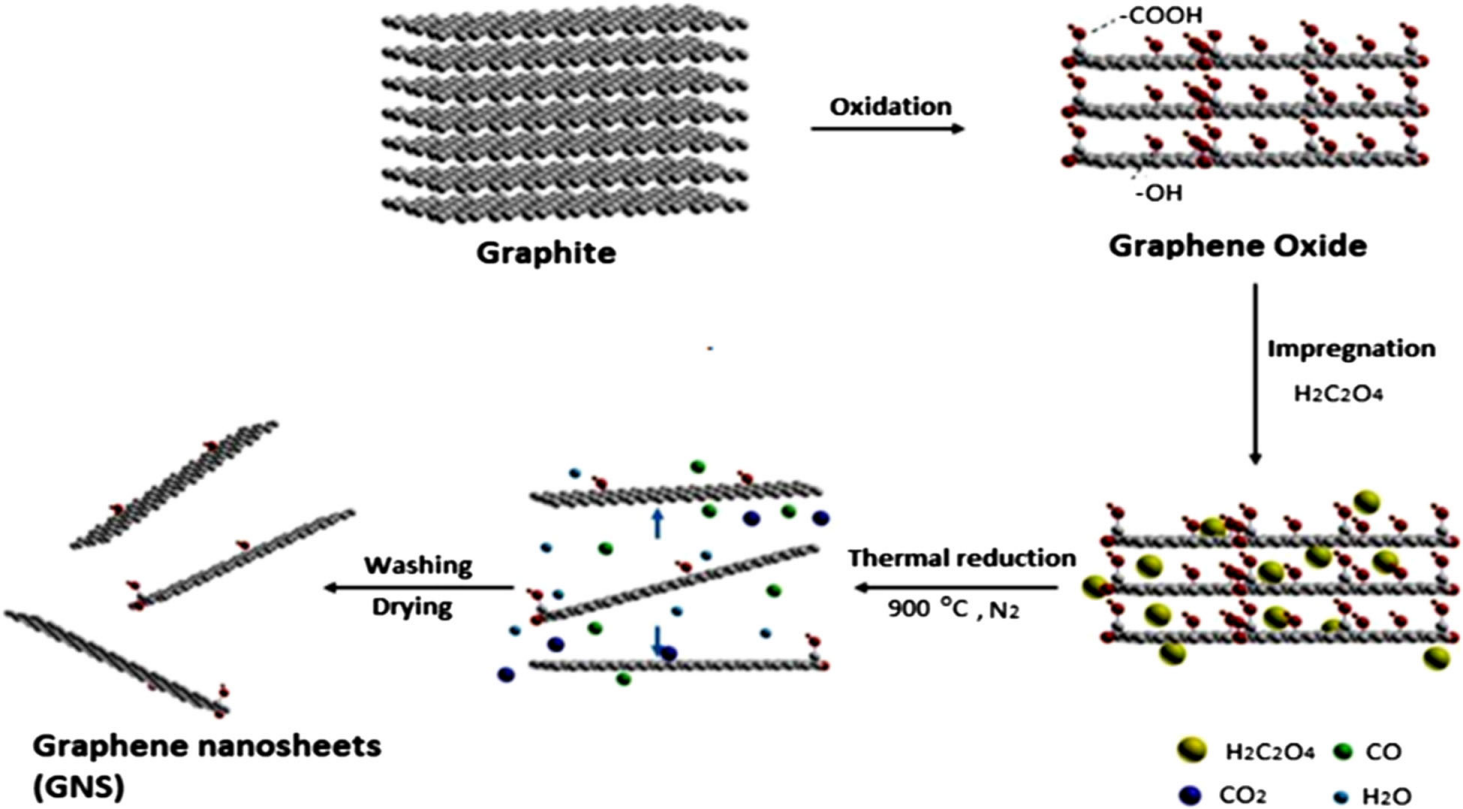
illustration of the preparation of graphite oxide to graphene nanosheets (GNS) by using oxalic acid [143]

**Fig. 5:**
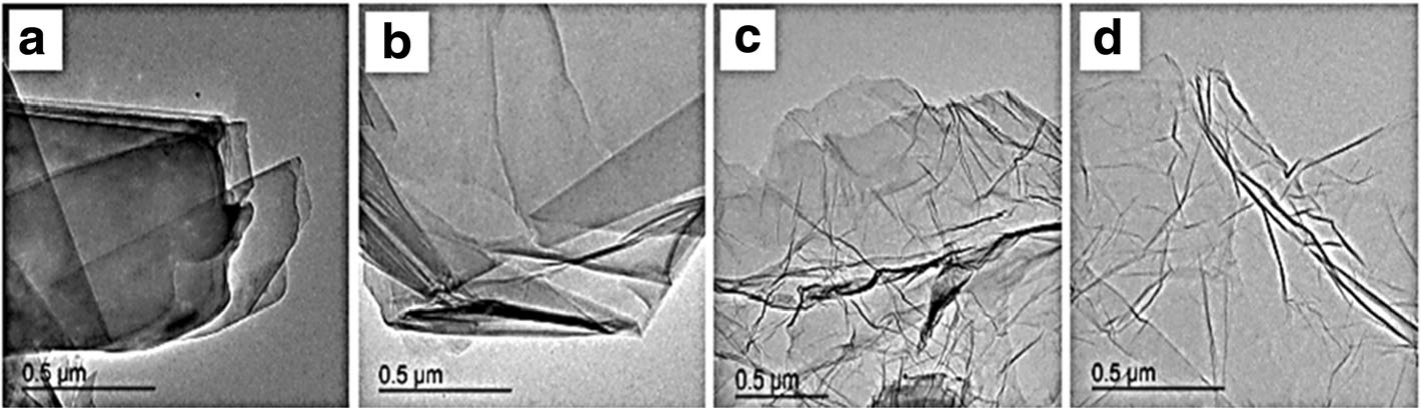
TEM images of graphite (**a**), GO (**b**), GN-900 (**c**), and GN-900-C [143]

Graphene has many extraordinary properties; it exists as a two-dimensional carbon (2-D) form, which is called a crystalline allotrope, one-atom-thick planar flat sheet of sp2 tightly bonded carbon atoms with a thickness of 0.34 nm. Its carbon atoms are packed in a regular atomic-scale chicken wire (hexagonal) pattern [92, 119]. The theoretical specific surface area of graphene is 2630 m^2^ g^−1^, which is much larger than that of carbon black (typically less than 900 m^2^ g^−1^) and carbon nanotubes (100 to 1000 m^2^ g^−1^) and similar to that of activated carbon [130]. Graphene has high potential as a metal support [131, 132, 133] [33] due to its high surface area [134] for better catalyst/metal dispersion [135], high electrical conductivity [136], and good thermal properties [137, 138]. Moreover, the functionality of graphene support can be modified by changing it surface structure, and hence contribute to its potential applications, such as in fuel cells, energy storage, electrochemistry, supercapacitors, and batteries [138–142]. Figure 4 illustrates the preparation steps to obtain the graphene nanosheets (GNS), while Fig. 5 shows their TEM images [143]. It can be clearly observed that the thickness of the GO with many typical wrinkles obviously decreases compared to graphite. This can be explained by the presence of the rich oxygen-containing functional groups over the surface of GO [132]. Besides, both resulting GN-900 and GN-900-C contained of a large size of nanosheets structure, but the GN-900-C comprised more transparent than the GN-900.

The discovery of graphene sheets began around 2000 by mechanical extracting process from 3D graphite source [133]. Graphene can be obtained by several synthesis methods such as hydrothermal [144], chemical reduction [143], chemical vapor deposition, and electrochemical. Ma et al. [145] enhanced the electrocatalytic activity of Pt nanoparticles by supporting the Pt nanoparticles on functionalized graphene for DMFC. Functionalized graphene was prepared by methyl viologen (MV) and Pt/MV–rGO electrocatalyst was synthesized by a facile wet chemical method. They also reported that the higher catalytic activity of Pt/MV–RGO was attributed to the synergetic effect between MV and rGO.

Meanwhile, Zhang et al. [146] modified the graphene support with graphene nanosheets through Hummer’s method, followed by polymerization of aniline (as nitrogen source). The TEM images for Pt/NCL-RGO and Pt/RGO electrocatalysts show that the aggregation between separated graphene sheets was decreased by nitrogen-doped carbon layer (NCL), leading to a better dispersion of the Pt catalyst on the graphene nanosheets support and better electroactivity and stability toward methanol electrooxidation (MOR). Presence of NCL successfully prevented the aggregation of graphene nanosheets as the Pt nanoparticles supporting material.

**Fig. 6:**
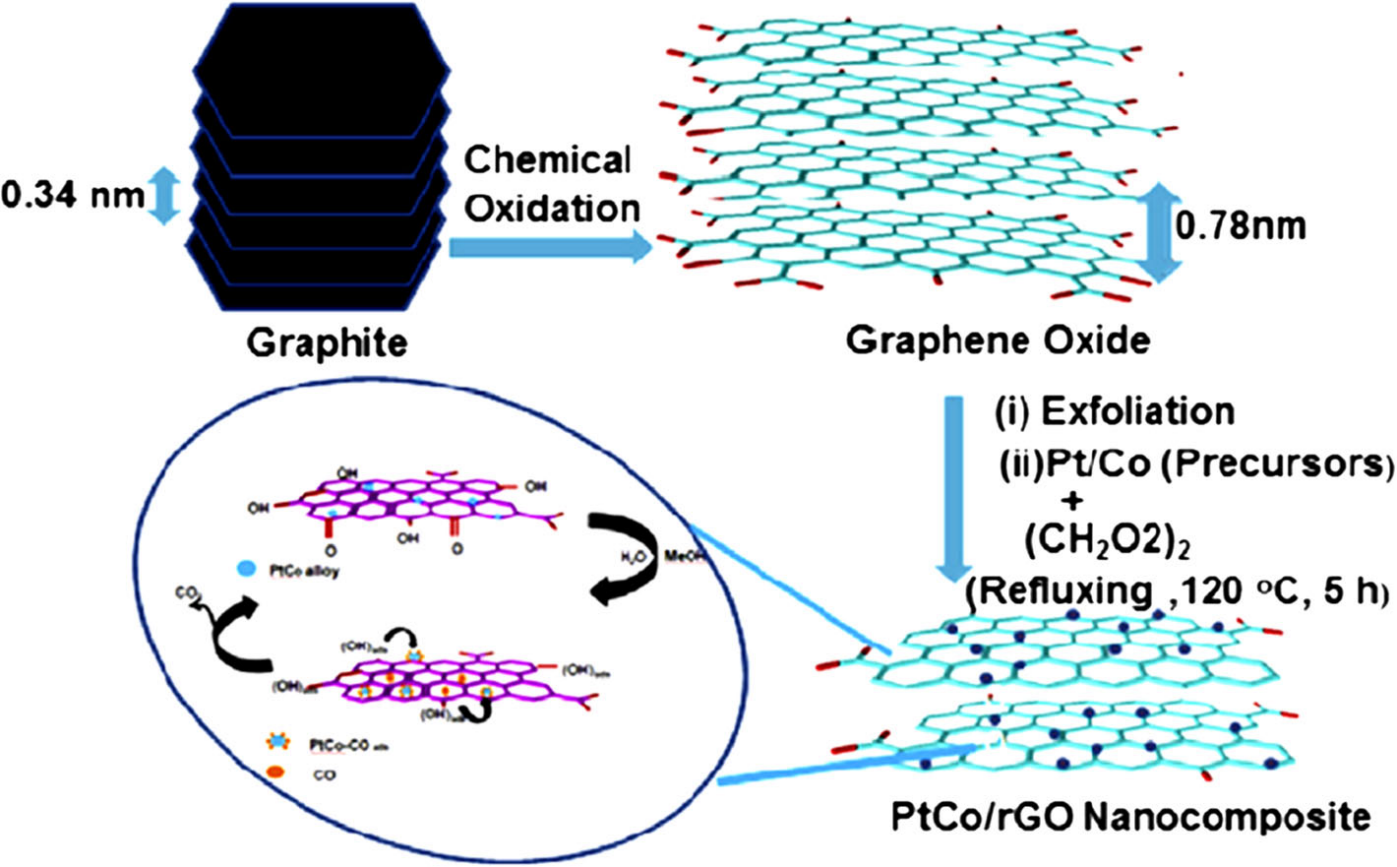
Illustrates the schematic formation of graphene supported Pt-Co catalyst [51]

In 2011, Qiu et al. [135] successfully synthesized nanometer-sized Pt catalyst via sodium borohydride reduction method with an average particle size of only 4.6 nm. These Pt nanoparticles showed an even dispersion of Pt catalyst on graphene oxide support and very high electrocatalytic activity toward MOR by controlling the percent deposition of Pt loaded on the graphene. In another study conducted by Ojani et al. [147], for synthesized Pt-Co/graphene electrocatalyst, it was shown that graphene nanosheets improved the electrocatalytic behavior and long-term stability of the electrode. In addition, the Pt-Co/G/GC electrocatalyst showed great stability toward MOR. The catalytic performance toward MOR can also be improved by using cobalt core–platinum shell nanoparticles supported on surface functionalized graphene [148]. This enhanced catalytic activity could be attributed to the poly (diallyldimethylammonium chloride) (PDDA) that plays a crucial role for dispersion and stabilization of Co@Pt catalyst on graphene support. PDDA-functionalized graphene provided the higher electrochemical active surface area [149, 150]. Huang et al. [138] also studied a PtCo-graphene electrocatalyst with outstanding catalytic performance and high CO tolerance toward the MOR, which far outperformed Pt-graphene and PtCo-MWCNT electrocatalysts with the same ratio of Pt and carbon content. Figure 4 shows the formation of a graphene-PtCo catalyst prepared from a graphite source. Sharma et al. [57] synthesized Pt/reduced graphene oxide (Pt/RGO) electrocatalyst using a microwave-assisted polyol process, which sped up the reduction of GO and formation of Pt nanocrystals. They compared Pt/RGO to a commercial carbon support (Pt/C), which exhibited high CO tolerance, high electrochemically active surface area, and high electrocatalytic activity for the MOR. In a previous study, Zhao et al. [139] reported that the unique 3D-structured Pt/C/graphene aerogel (Pt/C/GA) exhibited greater stability toward MOR with no decrease in electrocatalytic activity. Moreover, the Pt/C/graphene aerogel also exhibited significantly higher stability to scavenge crossover methanol at high potential in an acidic solution compared with the commercial Pt/C electrocatalyst. At the initial catalytic stage, the Pt/C electrocatalyst lost approximately 40% after 1000 CV cycles. In contrast, the Pt/C/graphene aerogel only lost 16% of the initial catalytic activity. After 200 cycles of CV, the current density of Pt/C/graphene aerogel was much higher with a remarkably higher stability than that of Pt/C electrocatalyst. Meanwhile, Yan et al. [151] demonstrated highly active mesoporous graphene-like nanobowls supported Pt catalyst with high surface area of 1091 m^2^ g^−1^, high pore volume of 2.7 cm^3^ g^−1^, and average pore diameter of 9.8 nm obtained by applying a template synthesis method. In addition, the Pt/graphene bowls also achieved high performance toward MOR with a current density value of 2075 mA mg_Pt_^−1^, which was 2.87 times higher than that of commercial Pt/C (723 mA mg_Pt_^−1^). The onset potential for the Pt/graphene bowls toward methanol electrooxidation was negatively shifted by approximately 160 mV compared with that to the latter and showed CO resistance. Figure 6 shows the proposed schematic for the formation of PtCo catalyst on reduced-GO (rGO) support [51]. It is described that the formation of graphene oxide nanosheets from oxidation of graphite powder leads to the increase in interlayer “d” spacing of stacked graphitic sheets from 0.34 to 0.78 nm due to the presence of various oxygen-containing functional groups. The oxygen-containing functional groups act as anchor sites for the well-dispersed Pt and PtCo nanoparticles on rGO sheets, and used for efficient electrooxidation of methanol.

We can conclude that the reduce graphene oxide (rGO), graphene, modified graphene as supporting material exhibited high electrocatalytic activity toward methanol electrooxidation process. A lot of studies have been reported related to the particle size distribution and size, morphologies, and catalytic activities of Pt and Pt alloys using graphene as supporting material, which showed great improvement in fuel cell performance as mentioned and discussed above. Thus, graphene support can be further studied for better fuel cell performance.

### Multiwall Carbon Nanotube and Single-Wall Carbon Nanotube Support

**Fig. 7:**
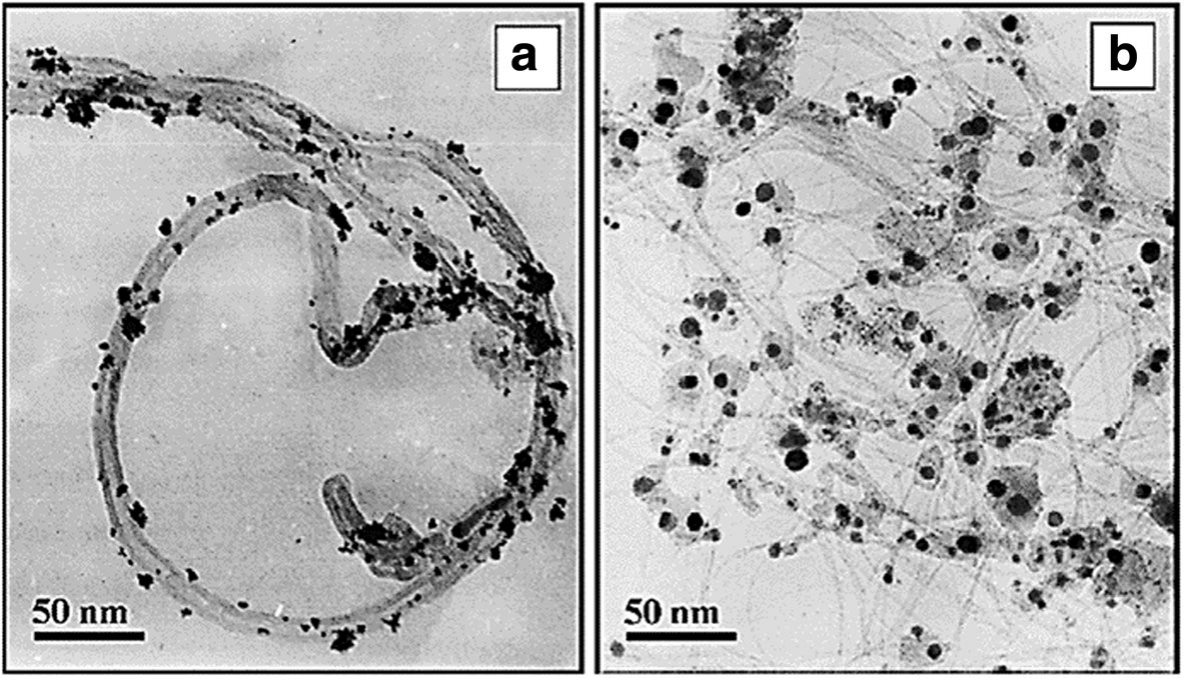
TEM images for the Pt on MWCNT(**a**) and SWCNT (**b**) [156]

Several years ago, Jha et al. [140] prepared multiwall carbon nanotube (MWCNTs) via chemical vapor deposition using an AB_3_ alloy hydride catalyst. Platinum-supported MWCNT (Pt/MWCNT) and platinum-ruthenium-supported MWCNT (Pt-Ru/MWCNT) electrocatalysts were prepared by chemical reduction. The performance of these electrodes was studied at different temperatures, and the results demonstrated a very high power density of 39.3 mW cm^−2^ at a current density of 130 mA cm^−2^, which could be attributed to the dispersion and accessibility of the MWCNT support and Pt-Ru in the electrocatalyst mixture for the methanol oxidation reaction. This was also done by other researchers that using different catalyst supported MWCNT for DMFC system [152–155]. Meanwhile, Wu and Xu [156] compared MWCNT-supported Pt and single-wall carbon nanotube (SWCNT)-supported Pt. Figure 7 shows that the TEM images of Pt catalyst was deposited on MWNT and SWNT electrodes through the electrodeposition technique. The Pt particles in Pt-SWNT (Fig. 7b) looked closer contact with the network of entangled and branched bundles of SWNT support, and the shape is closer to highly exposed sphere. The benefits of the SWCNT support are due to its greater electrochemical surface-active area and easier charge transfer at the electrode/electrolyte interface because of the graphitic crystallinity structure, rich amount of oxygen-containing surface functional groups, and highly mesoporous and unique 3D-structure of SWNT. The electrodeposition technique carried out by them contributed to higher utilization and more uniform dispersion of Pt particles on its support.

Then, Wang et al. [157] reported the high performance of modified PtAu/MWCNT@TiO_2_ electrocatalyst prepared via deposition-UV-photoreduction for DMFC, which also exhibited high CO tolerance. Zhao et al. [126] studied 3D flower-like platinum-ruthenium (PtRu) and platinum-ruthenium-nickel (PtRuNi) alloy nanoparticle clusters on MWCNTs prepared via a three-step process, and the best ratios obtained from their experiments for the PtRu and PtRuNi alloys were 8:2 and 8:1:1, respectively. Another group, i.e., Zhao et al. [158], found a higher current density toward MOR and better activity for MWCNT-supported PtWC compared with Pt/C electrocatalyst. These results were attributed to the factors of the synergistic effect between the Pt catalyst and the WC component, high CO tolerance from the bifunctional effect of the Pt catalyst and the WC component, and strong interaction between metals and WC in the electrocatalyst composite.

As a summary, both of MWCNT and SWNT support in terms of structural, surface, and electrochemical properties have their own characteristics as supporting material that remarkably enhanced their performance in catalysis of methanol oxidation process. However, as a comparison, SWCNT possess a high degree of graphitization, highly mesoporous 3D structure, and contain more oxygen-containing functional groups at its surface sites. In relation with these properties, the SWCNT exhibits a higher electrochemically accessible surface area and faster charge transfer rate at the electrode/electrolyte interface.

### Carbon Nanotube Support

**Fig. 8:**
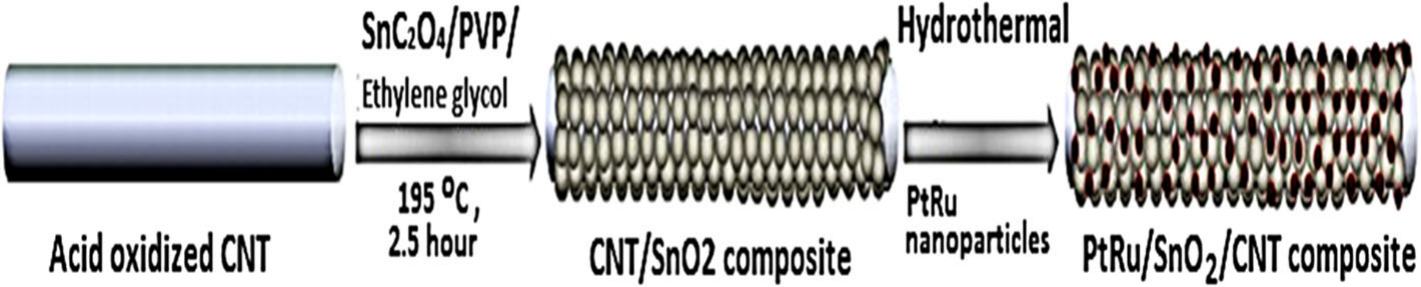
Illustrates the schematic diagram for the formation of PtRu/SnO2/CNT composite [128]

**Fig. 9:**
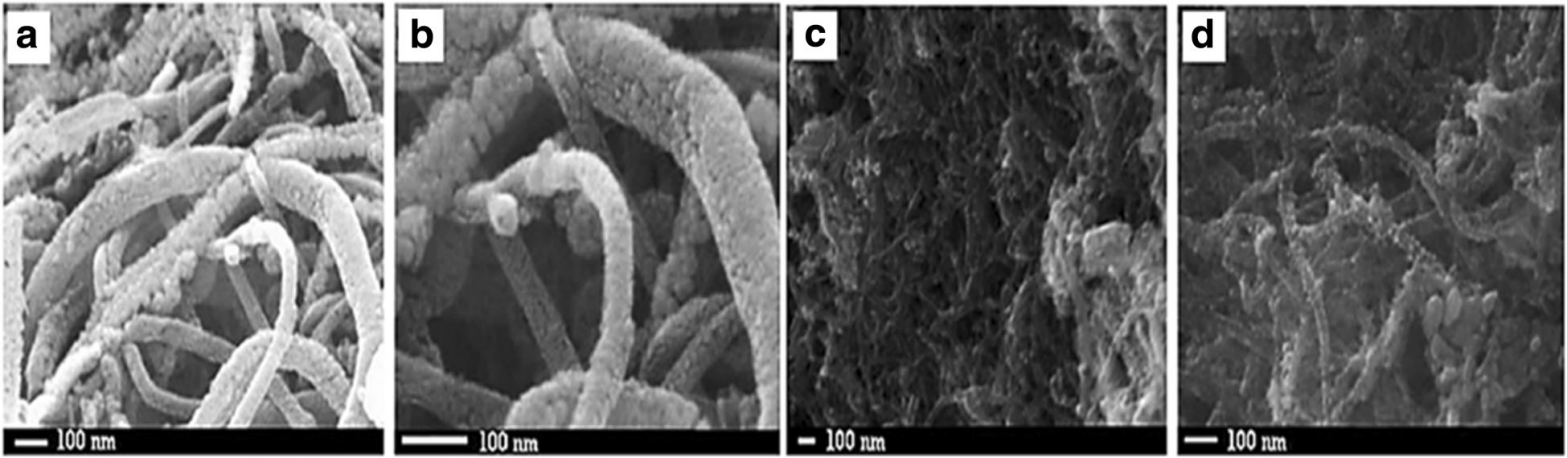
FESEM images of CNT/SnO2 composite support (**a**, **b**) and PtRu/SnO2/CNT composite electrocatalyst (**c**, **d**)

Wen et al. [144] proposed that carbon nanotubes (CNTs) support could improve fuel cell performance; for example, Pt can be fixed to the inner wall and the outer wall of CNTs and may cause improvement in the electrocatalytic properties of platinum-CNTs. Yoshitake et al. [159] proposed that fuel cells using CNTs as the catalyst support produced larger current densities. The addition of binary or other components to the electrocatalysts for methanol electrooxidation overcomes the problems related to catalyst poisoning caused by CO during the reaction. Therefore, new electrocatalyst carbon supports, such as carbon nanotubes [160, 161], are being actively developed to significantly improve fuel cell performance. Kakati et al. [128] successfully synthesis the PtRu on CNT/SnO_2_ for anode catalyst DMFC via hydrothermal process. It has been found that the presence of SnO_2_ provide a high durability property for the catalyst and the presence of SnO_2_ in the district of Pt could supply oxygen-containing functional groups for the removal of CO intermediate molecules from the Pt surface sites during electrooxidation of methanol. Generally, the decomposition methanol occurs at Pt surface sites; meanwhile, the decomposition of water occurs at SnO_2_ surface sites to form oxygen-containing species which then react with CO intermediate molecules. However, as support material, the conductivity property of SnO_2_ still needs to be enhanced. Kakati et al. [128] also proposed the schematic diagram of the formation of PtRu on CNT/SnO_2_ composite as shows in Fig. 8, and FESEM images of CNT/SnO_2_ composite support (a and b) and PtRu/SnO_2_/CNT composite electrocatalyst (c and d) in Fig. 9.

Chien et al. [127] proposed that the high catalytic performance of Pt-Ru/CNT for MOR can be attributed to the presence of CNT as the carbon support material with several factors: (i) the as-synthesized Pt-Ru/CNT electrocatalyst owns the ideal nanosized particles and composition to increase catalytic activity, (ii) the presence of functional group on the CNT surface results in high hydrophilicity of CNT, which produces better electrochemical reaction on the electrode area, and (iii) the high electronic conductivity of the CNT support lowers the resistance in MOR. Jeng et al. [150] prepared Pt-Ru/CNT electrocatalyst via a modified polyol with a PtRu composition ratio of 1:1, exhibiting high catalytic activity toward MOR and better performance than that of commercial PtRu/C. Show et al. [162] reported that Pt catalyst with a size of less than 10 nm can be obtained by dispersing the Pt particles on a CNT surface using the in-liquid plasma method, and excellent performance was demonstrated by the electrical power achieving 108 mW cm^−2^ [162]. The in-liquid plasma method was also used by Matsuda et al. [163] that can applied to obtain nanometer-sized Pt catalyst on support material that remarkably enhanced the fuel cell performance.

To be concluded, high electric conductivity, large surface area, excellent chemical and electrochemical stabilities, quasi one-dimensional structure, and good morphology as the supporting materials are the key factors of carbon nanotubes (CNTs) in enhancing the DMFC performance. In addition, carbon support materials such as CNTs which contribute a large effect on metal distribution and size have also been proven to be an essential to the electrocatalysts to achieve high catalytic activity during methanol oxidation process.

### Carbon Nanofiber Support

Steigerwalt et al. [164] reported the successful synthesis of PtRu alloy that was widely dispersed on a graphene carbon nanofiber (CNF) support as an electrocatalyst in DMFC. The catalytic activity was enhanced by ~ 50% relative to that recorded for an unsupported PtRu colloid anode electrocatalyst. Meanwhile, Wang et al. [152] reported that Pt/CNF nanocomposites obtained by the reduction of hexachloroplatinic acid (H_2_PtCl_6_) precursor with formic acid (HCOOH) in aqueous solution containing electrospun CNFs at room temperature showed a higher current density than other prepared Pt/CNFs and was approximately 3.5 times greater than that of the E-TEK Pt/C electrocatalyst. Another research carried out by Giorgi et al. [153] described a CNF and bimetallic PtAu electrode with a single layer and both diffusive and catalytic functions using a decreased noble metal amount (approximately five times less) with a consequent large cost reduction. In addition, the bifunctional electrocatalytic properties were also active for the MOR on the PtAu nanoparticle catalysts [154]. Calderón et al. [155] reported PtRu/CNF prepared via reduction using sodium borohydride (NaBH_4_), methanol, and formate ions. This electrocatalyst synthesized by SFM was heat-treated (denoted as SFM TT), which improved its electrocatalytic activity during MOR. Later, Maiyalagan [165] reported that the addition of silicotungstic acid acted as a stabilizer for the PtRu particles on CNT support. The PtRu-supported CNT was prepared by microwave heating of an ethylene glycol (EG) solution of STA, H_2_PtCl_6_.6H_2_O (as Pt precursor), and RuCl_3_.xH_2_O (as Ru precursor) with CNF suspended in the solution. The Pt and Ru precursors were loaded on CNF by conventional impregnation method. The results revealed that the PtRu nanoparticles are uniformly dispersed on carbon nanofiber support, with an average particle size of 3.9 nm enhanced the catalytic activity toward methanol electrooxidation. As a conclusion, the carbon nanotubes supporting material with high electronic conductivity and high surface area gives an advantage of better dispersion for the Pt or Pt alloys deposition. The higher the surface area of supporting material can reduce the agglomeration of metal particles on it, thus can produce better catalyst morphology for better fuel cell performance.

### Mesoporous Carbon Support

Mesoporous carbon (MPC) support is another ideal candidate as an electrocatalyst support material in DMFC and fuel cell. Generally, mesoporous carbons are divided into two classes based on their structures which are ordered mesoporous carbons (OMCs), with highly ordered pore structure and uniform pore size, nonordered mesoporous carbons with irregular pores. Other than that, OPC can be produced by using high quality of SBA-15 silica and sucrose as carbon source template. To prepare the high quality of SBA-15 SBA-15 sample, triblock copolymer, EO20-PO70EO20 (Pluronic P123, BASF), as the surfactant and tetraethyl orthosilicate (TEOS, 98%, Acros) as the silica source are used, as reported by literature [166–168]. The synthesis of MPC starts from synthesis of SBA-15, followed by calcination process.

A well-dispersed and ultralow Pt catalyst (PtFe) supported on ordered mesoporous carbon (OMC) was prepared via a simple route and showed superior catalytic activity. The PtFe alloy with a size range of 3–5 nm was homogeneously dispersed on the CMS with a very high specific surface area of more than 1000 m^2^ g^−1^ [169]. The incorporation of Fe was discussed in the previous section (“Performance of Various Types of Pt-Based Catalysts” section and “Performance of Pt-Based Alloys” section). The high specific surface area of mesoporous carbon support can be produced by carbonization process of a resorcinol-formaldehyde polymer with a cationic polyelectrolyte as a soft template [160]. The performance of Pt/MPC also related to the synthesis/preparation method as done by Kuppan and Selvam. Kuppan and Selvam [167] synthesized four type of Pt/mesoporous carbon by using different reducing agent which are NaBH_4_, EG, hydrogen, and paraformaldehyde. From there, the Pt/mesoporous carbon synthesized using paraformaldehyde as reducing agent for showed highest current density. The highest in catalytic was attributed to the use of paraformaldehyde that gives the smallest Pt particle size (4.5 nm), and the highest ECSA (84 m^2^/g) belongs to Pt/mesoporous carbon.

Wang et al. [161] synthesized a Pt@WC/OMC electrocatalyst composite, in which the composite was platinized using a pulsed microwave-assisted polyol technique. The OMC produced in this synthesis exhibited high surface area property. The Pt@WC/OMC electrocatalyst also showed high activity, desirable stability, and CO tolerance toward MOR. In another work done by Zhang et al. [170], the ordered CMS had a unique hierarchical nanostructure (with a 3-D structure) with ordered large mesopores and macropores that facilitated the dispersion of Pt nanoparticles and rapid mass transport during the reactions.

To maximize the use of Pt particles, the support materials should have uniform dispersion, high utilization efficiency, and desirable activity and stability. Moreover, the good supporting materials must be suitable for surface chemistry, high loading of Pt dispersion, and some functional roles. Additionally, based on the previous studies as discussed above, the ordered mesoporous carbons with large pore sizes are highly desirable for fast mass transfer and, thus, enhance the catalytic activity especially in the reaction involve large reactants molecules.

### Carbon Black

Carbon black (CB) is one of the commercial carbon support that has been used till now. There are many types of CB such as Vulcan XC-72, Black Pearl 2000, Denka Black, Shawinigan Black, Ketjen EC-300J, etc. [171, 172]. CB is commonly used as a carbon support material for electrocatalysts because it possesses high porosity properties, which make it suitable as a potential support material for the catalyst layer in PEMFCs and DMFCs as reported in provided literatures [173–180]. The comparison of the several carbon black support was reported by Wang et al. [181] who investigated the effect on DMFC performance using several types of carbon black such as Vulcan XC-72R, Ketjen Black EC 300J, and Black Pearls 2000 carbon black as additives/support for the Pt cathode catalyst. From the experiments, the results showed that Ketjen Black EC 300J was the most useful carbon support for increasing the electrochemical surface area and DMFC performance of the cathode catalyst.

Nowadays, CB is commercial carbon support for many fuel cell systems. Generally, it is used for the comparison with new or modified catalyst [125]. The following Table 3 summarizes the commercial carbon black and its properties for fuel cell application. There are so many modifications among carbon support materials and development of new carbon support for enhance fuel cell performance; however, commercial carbon black still is used in many fuel cell applications especially for the comparison with new or modified catalyst.

**Table 3:** Properties of carbon black sources

Types of carbon black	Supplier	Surface area	Method of production	Particle size	References
Vulcan XC-72	Cabot	~ 250	Furnace Black	20–50 nm	[198]
Denka Black	Denka	~ 65	Acetylene Black	40 nm	[199]
Ketjen EC-600 JD	Akzo Nobel	~ 1300	Furnace Black	30 nm	[200]
Black Pearl 2000	Cabot	~ 1500	Furnace Black	15 nm	[201]
Ketjen EC-300 J	Akzo Nobel	~ 800	Furnace Black	30 nm	[202]
Shawinigan Black	Chevron	~ 80	Acetylene Black	40–50 nm	[199]
Conductex 975 Ultra	Columbian	~ 250	Furnace Black	24 nm	[201]
3250/3750/3950	Mitsubishi	240/800/1500	–	28 nm/28 nm/16 nm	[202]

### Carbon Nanocoils

Celorrio et al. [182] proposed carbon nanocoils (CNCs) as a PtRu support in their experiment, indicating that the electrocatalyst performance was strongly dependent on the synthesis method. CNC-supported electrocatalysts showed better electrochemical behavior than E-TEK electrocatalysts, and better electrocatalytic behaviors toward CO and methanol oxidation were achieved using CNC as a support material [182]. Sevilla et al. obtained highly graphitic CNCs from the catalytic graphitization of carbon spherules via the hydrothermal treatment of different saccharides which are sucrose, glucose, and starch [183]. They demonstrated that the high electrocatalytic activity of the CNCs is due to the combination of good electrical conductivity of their graphitic structure and high porosity property, which allows much less diffusional resistance of reactants/products. Two years later, Sevilla et al. [184] reported highly dispersed Pt nanoparticles on graphitic CNCs with diameters in the range of 3.0–3.3 nm and a very fine particle size distribution. The electrocatalyst possessed large active Pt surface area (up to 85 m^2^ g^−1^ Pt), high catalytic activity toward MOR (up to 201 A g^−1^ Pt), and high resistance against oxidation, which was noticeably greater than that of the Pt/Vulcan electrocatalyst. Celorrio et al. [185] obtained Pt/CNC electrocatalysts via the impregnation method, which showed that a combination of Pt and CNCs facilitated the CO oxidation process.

## Conductive Polymer Supports

Choi et al. [186] synthesized PtRu alloy nanoparticles with two types of conducting polymers, i.e., poly(*N*-vinyl carbazole) and poly(9-(4-vinyl-phenyl)carbazole), as the anode electrodes. Electrochemical and DMFC tests showed that these nanocomposite electrocatalysts were beneficial in a DMFC system, but their catalytic performance was still lower than that of a carbon supported electrode. Thus, they suggested that higher electrical conductivity of the polymer and lower catalyst loss are required in nanocomposite electrodes to achieve better performance in a DMFC. Choi et al. [171] and Kim et al. [172] prepared polyaniline (PANi) as a support material for PtRu catalyst in a DMFC system. PANi is a group of conductive polymers with high electronic conductivity and a methanol oxidation current similar to that of carbon-supported PtRu catalyst. Then, Kim et al. [172] conducted catalytic tests to compare PtRu/PANi support with PtRu/carbon support, showing that the enhanced catalytic activity of PtRu/PANi was due to (i) the high electrical conductivity of the polyaniline support, (ii) the increase of electrochemical surface area of the prepared electrocatalyst, and (iii) the higher ion diffusion behavior. In another study, Amani et al. [74] synthesized PtSn supported by C-PANI as an electrocatalyst with different Pt:Sn atomic ratios using the impregnation method. The PtSn/C-PANI electrocatalyst with a ratio of 30:70 showed outstanding performance in the methanol electrooxidation, and the current density was approximately 40% higher than PtRu/C and 50% higher than Pt/C-PANi. The CO tolerance and stability were improved compared to that of PtRu/C, and the methanol crossover was reduced. Yaldagard et al. [173] studied the electrocatalytic performance of Pt/PANi/WC/C electrocatalyst for methanol electrooxidation (MOR) and oxygen electro-reduction (ORR), and it exhibited higher MOR activity, high CO resistance, and improved stability compared to Pt/C electrocatalyst in the presence of methanol.

Wu et al. [174] presented polypyrrole nanowire networks (PPNNs) as the anodic microporous layers (MPLs) of passive DMFC. In passive DMFC system, the novel MPL achieved a 28.3% increase in the power density from 33.9 to 43.5 mW cm^−2^ compared with the conventional layer with a similar PtRu (1:1). The high performance was due to the presence of PPNNs, which expressively improved the catalyst utilization and mass transfer of methanol on the anode. Besides, Selvaraj and Alagar [175] prepared Pt-Ru nanoparticle-decorated polypyrrole/multiwalled carbon nanotubes (Ppy/CNT) via the in situ polymerization of Ppy on CNTs containing ammonium peroxydisulphate (NH_4_)S_2_O_8_ as an oxidizing agent at the temperature range of 0–5 °C, followed by deposition of Pt particles on PPy-CNT composite films via chemical reduction to produce Pt/PPy-CNT. It was found that the PtRu particles deposited on PPy–CNT composite films exhibited higher catalytic activity and stability toward MOR compared to Pt/PPy-CNT. So far, the investigation on polymer as supporting materials is not much as carbon support materials. From aspect as supporting materials, the performance of polymer support was not good/excellent as carbon support. Further studies are needed in the future for better electrocatalytic activity and DMFC performance.

## Problems and Limitations of Using Pt for DMFC Systems

There are two major challenges in the development of new DMFC catalysts: (i) performance, including the catalytic activity, reliability, and durability; and (ii) catalyst cost reduction. Two major problems arise in DMFC when using pure Pt alone as the anode catalysts: (1) slower kinetics oxidation of methanol, even on some state-of-the-art anode catalysts, and methanol crossover through the membrane, which not only lowers cathode performance but also reduces fuel efficiency. To develop successful fuel cell technology, including DMFC technology, new catalysts must be investigated to improve the performance and reduce the cost. Reduction of the catalyst cost remains a major challenge. Currently, platinum is one of the most effective electrocatalysts for DMFC due to its high catalytic activity for methanol oxidation, but because it is a precious metal, platinum usage is challenging and limited [176, 177]. Therefore, many scientists have attempted to find materials that can behave like Pt catalysts. One problem with the MOR in DMFCs is that CO is produced as an intermediate reaction product when using Pt catalyst and has strong binding energy on platinum particles, poisoning the active sites of the platinum surface area [58]. Therefore, CO must be removed by oxidizing it from the Pt surface using another material with high resistance to CO poisoning. For example, Hwu et al. proposed Pt-modified WC catalyst that has remarkable resistance to CO poisoning [178]. On the other hand, they also suggested that CO tolerance originates from the lower CO desorption temperature on pure and Pt-modified WC compared to pure Pt.

**Fig. 10:**
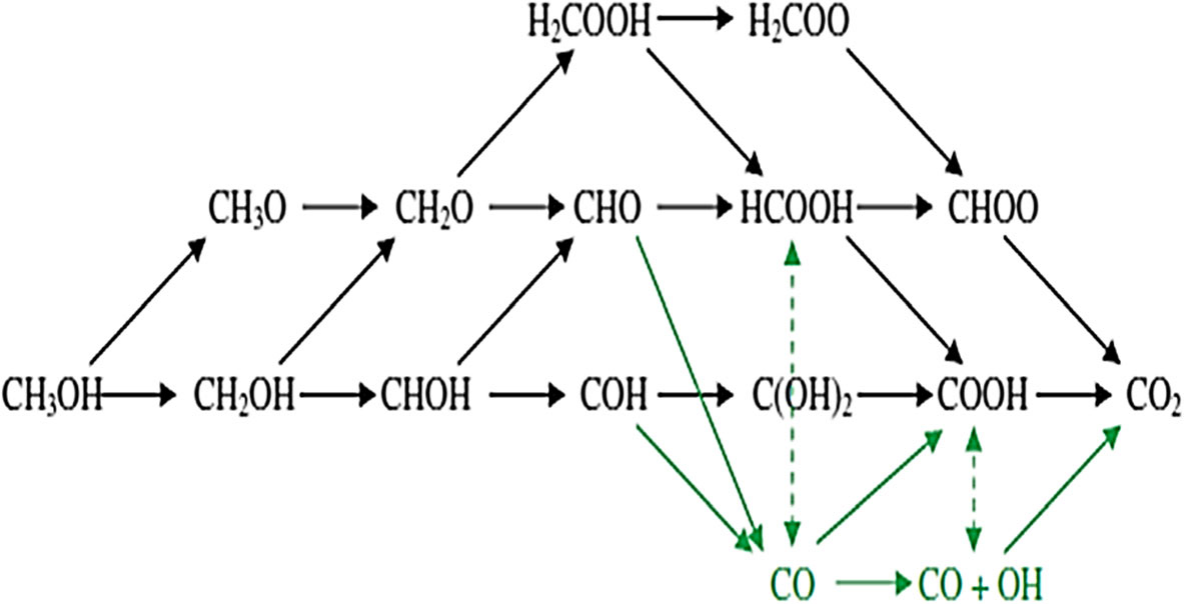
Schematic of the reaction paths and possible intermediates molecules considered in methanol electrooxidation [237]

There are many solutions that can be applied to reduce the cost of Pt, overcome or minimize the formation of CO species during methanol oxidation, and increase the kinetics of methanol oxidation, such as alloying with other metals or transition metals, the incorporation of metals, metal nitrides, and metal oxides and the use of carbon supports as discussed in this paper. However, to overcome this problem, we need to understand the formation of CO on Pt sites particle, and understanding of the mechanism of the anode reaction in DMFCs. Unfortunately, it has limited amount of mechanistic insight to be studied, because this reactions involve complex mechanism path with many possible intermediate molecules and also competing reaction pathways [179]. For Pt catalytic mechanism, it has been suggested by a direct reaction path. Unfortunately, the use of Pt on other metals has limited mechanistic information available. Figure 10 represents the reaction path for methanol electrooxidation and their possible intermediates molecules formed during the process. Black arrows show direct path, while green arrows show the indirect mechanism for CO_2_ formation as a final product. In the direct mechanism, the reaction path does not involve a CO intermediate, and CO_2_ is formed directly from methanol. In contrast, indirect mechanism forming a CO intermediate molecule and subsequently it is oxidized to CO_2_ product. Notably, CO is the most stable molecule of all the intermediates on Pt during MOR. The stability of CO causes it to be a main reason for the extensive CO poisoning problem that is often found on Pt catalyst.

First step in the mechanism of methanol decomposition reaction on Pt is the activation of methanol molecule. It can take place via hydrogen abstraction from either the carbon or the oxygen atoms. Further step, hydrogen abstraction creates formaldehyde (CH_2_O) or hydroxymethylene (CHOH), followed by formyl (CHO) or COH. In the direct mechanism, instead of stripping off the final hydrogen from CHO or COH molecule to CO, a water molecule will release a proton/electron pair and resulting to OH species that can further bind with the carbonaceous species to form dihydroxycarbene (C(OH)_2_) or formic acid (HCOOH). This step is called hydroxyl addition process. The next step is followed by dehydrogenation to form either formate (HCOO) or carboxyl (COOH) molecule, with subsequent dehydrogenation to form CO_2_ as the final product of reaction. In addition, an alternative direct mechanism involve the stripping of a proton/electron pair from water and addition of the resulting hydroxyl to CH_2_O, subsequently to H_2_COOH, which then undergoes dehydrogenation to form HCOOH or dioxymethylene (H_s_COO). The H_s_COO molecule can then undergoes dehydrogenation to HCOO and finally to CO_2_. Besides, in the indirect mechanism, CHO or COH species are directly dehydrogenated to CO. Water is dissociated separately on the surface to form OH, and the two surface species react together to form CO_2_ gas in a way similar to the water-gas-shift reaction [187]. This indirect mechanism occurs because less energy is required to form CO than CO_2_. Strong adsorbed CO intermediate form on the Pt surface sites revealed a major problem at the anode site of DMFC. Formation of this intermediate species can cause deactivation Pt catalyst. Furthermore, the rate of kinetic methanol oxidation for DMFC is slower. Therefore, to increase the resistance of Pt catalyst to CO poisoning on the electrodes, Pt alloy or hybrids, such as PtRu, PtSn, PtMO, PtPb, PtFe, PtCo, PtNi, PtRuOs, PtRuMo, PtRuSn, PtRuNi, etc. (as mentioned and discussed in “Performance of various types of Pt-based catalysts” section), are usually employed as electrocatalyst materials on DMFC anodes. Addition/incorporation of these alloys to Pt can prevent the adoption of CO on Pt surface by decreasing the oxidation overpotential of the anode [84].

## Conclusion and Prospects

Great progress has been made in recent years in the development and optimization of new catalysts using Pt-based catalysts and carbon and conductive polymer supports for DMFC anode catalyst. Some new carbon materials, such as nano- or mesostructured carbons, have been demonstrated as highly potential catalyst support materials, although their applications face challenges in terms of synthesis, metal loading, and electrode preparation. The combination of platinum as the best metal catalyst for DMFC and an excellent carbon support could produce breakthroughs in the investigation of a new DMFC anode catalyst in the future. Since platinum is an expensive metal, it is necessary to reduce the amount of Pt used in the electrocatalyst. Therefore, this paper presented more than 100 studies on the electrocatalytic activity and performance related to Pt-based electrocatalysts and various carbon and conductive polymer supports. The main problems related to the platinum electrocatalyst, such as carbon monoxide formation during the methanol oxidation reaction and the poor kinetics of methanol oxidation, could be overcome using additional materials and various supports, as reported in the research presented in this paper.

Many studies conducted in the recent years to reduce the loading amount of Pt catalyst and to increase the percentage utilization efficiency, and hence, enhance the electrocatalytic activity of Pt toward the oxygen reduction reaction (ORR) and methanol electrooxidation reaction (MOR), were discussed in this paper. Pt has been alloyed with many transition metals such as Fe, Co, Ni, Ir, Ru, Rh, and Pd, resulting in higher catalytic activity for the DMFC system. The incorporation of these materials also resulted in good dispersion on the carbon and polymer supports, which showed higher performance in the DMFC test compared to the use of Pt metal alone. Various carbon support sources, namely activated carbon (AC), carbon black (CB), multiwall carbon nanotubes (MWCNTs), carbon nanofibers (CNFs), carbon nanotubes (CNTs), graphene, and conductive polymer supports, have been used with Pt-based catalysts to improve their catalytic performance. Additionally, Pt-based alloy catalysts have been designed as hollow mesoporous PtNi, nanowire PtRu, and nanodendritic PtRh, which showed improved electrocatalytic activity and superior electrocatalytic performance. Meanwhile, 3-D Pt/C/graphene aerogel demonstrated enhanced stability toward methanol electrooxidation. The work performed by researchers showed that the electrocatalytic activities of nanoparticles Pt alloy catalysts depend on several factors such as the synthesis method, condition of experiments (such as temperature and pH), alloy composition/ratio, precursors, and thermal treatment. For the future study, it should be extended to the optimization of the geometry and structure of previous studies that revealed active Pt alloys can increase their electrocatalytic activity and stability and the application of support materials for fuel cell applications. For example, current research that have been done by Liu et al. 2017 [188] shows the excellent performance of platinum. From theoretical calculations, it revealed that the main effective sites on platinum single atom electrocatalysts are single-pyridinic-nitrogen-atom-anchored single-platinum-atom centers, which ascribed to the tolerant CO in MOR. They also suggested that carbon black supported used together with Pt single atom is effective in cost, efficient, and durable electrocatalyst for fuel cell application. According to the above study, herein, we can conclude that the modification on structure and morphology of precious metal such as platinum could also remarkably increase the performance of electrocatalyst, but in the same time can reduce the overall cost of fuel cell for commercialization.

To improve the morphologies of Pt and Pt alloys, carbon support material also need further study. Nanoporous metals become an interesting part of catalyst to be studied for fuel cell application. It is determined very suitable for fuel cell catalysts because they possess high surface area, three-dimensional (3D) network structures with adjustable ligament/pore sizes suitable for mass transport, and electron conduction. Around 2017, Li et al. successfully carried out modification on Pt-Pd-Au trimetallic surface as cathode for oxygen reduction reaction [189]. The surface evolution of 3-D Pt-Pd-Au trimetallic greatly enhanced the ORR activity and highly stable as ORR catalyst. The modification of PtNi alloy also done by Li et al. 2016 [190] showed ultrafine jagged platinum nanowire with highly large ECSA that exhibits enhanced mass activity of ~ 50 times higher than state-of-the-art commercial Pt/C catalyst, while Bu et al. 2016 [191] reported highly uniform PtPb/Pt core/shell nanoplate with biaxially strain extremely active, stable for anodic oxidation reactions, and great performance compared to commercial Pt/C in both methanol oxidation reaction (MOR) and ethanol oxidation reaction (EOR). Since the nanostructured platinum becomes an efficient catalyst for fuel cells as well as various industrial chemical reactions. Thus, these modifications on surface of Pt particles electrocatalysts could also to be applied in MOR for future DMFC.

On the other hand, to reduce the consumption of the Pt catalysts, the modification of the carbon support is also another useful way. This not only improves the transport capacity of protons but also reduces the usage of Nafion, which can cut the cost of the fuel cell. Moreover, with regards to the carbon support for the ORR catalysis, the hydrophobic carbon support material is required to allow water (product) to be quickly removed from the catalyst surface sites, and oxygen (reactant) to access the active sites. In contrast, the MOR catalysis requires a certain degree of hydrophilic carbon support. It can be achieved by the modification of the carbon support materials. By combination of modified carbon support materials and development of new carbon support with Pt metal catalyst, it is possible to get an ideal electrocatalysts for direct methanol fuel cell technology. Combination of Pt metal with varied carbon supports with different specific surface areas, structures, pore sizes, electronic properties, and morphologies could be great catalyst to be studied for future DMFC.

Carbon support also influence the overall performance for DMFC. Vulcan XC-72R, which is a commercial carbon support, has a large surface area, appropriate particle size, and good electrical conductivity for good support. However, in the process of depositing metal particle on these support with loading of 40% or more, the particle size of metal increased quickly, which is a disadvantage for DMFC, because a higher metal loading is used to give a better performance. In addition, multiwalled carbon nanotubes (MWCNTs) and carbon nanofibers (CNFs) with relatively smaller surface area, large diameter, and high aspect ratio could be very difficult to deposit a catalyst with high loading metal (40% and more). Therefore, modification of MWCNTs and CNFs support must be done to improve its surface area, surface functional groups, and reduce the wall thickness to achieve outstanding performance for direct methanol fuel cell even though high loading metal catalyst is consumed. As well, a great and important part to be further studied in DMFC system is about the anode and cathode catalyst preparation approaches.

## Abbreviations

CB: Carbon black
CH_3_O: methoxy group
CNC: Carbon nano cage
CNF: Carbon nano fiber
CNT: Carbon nano tube
Co: Cobalt
Co: Cobalt
CO: Monoxide molecules
CO_2_: Carbon dioxide
DMFC: Direct methanol fuel cell
FC: Fuel cell
Fe: Iron
MOR: Methanol oxidation reaction
MPC: Mesoporous carbon
MWCNT: Multi wall carbon nanotube
Ni: Nickel
OMC: Ordered mesoporous carbon
ORR: Oxygen reduction reaction
PANi: Polyaniline
PEMFC: Proton exchange membrane fuel cell
Ppy: Polypyrrole
Pt: Platinum
Pt/MWCNT: Platinum-supported MWCNT
Pt-Ru/MWCNT: Platinum-ruthenium-supported MWCNT
Rh: Rhodium
Ru: Ruthenium
Sn: Sternum
SOFC: Solid oxide fuel cell
SWCNT: Single-wall carbon nanotube
TMN: Transition metal nitride
